# Supplementary material for: Immune-featured stromal niches associate with response to neoadjuvant immunotherapy in oral squamous cell carcinoma
Source: Cell Rep Med. 2025 Mar 18;6(3):102024. doi: 10.1016/j.xcrm.2025.102024 (PMC11970382; doi:10.1016/j.xcrm.2025.102024)
Supplement: Document S1. Figures S1–S10 and Tables S1 and S2 [file mmc1.pdf]

Cell Reports Medicine, Volume *6*

Supplemental information

Immune-featured stromal niches associate with response to neoadjuvant immunotherapy in oral squamous cell carcinoma

Yu-Tong Liu, Hai-Ming Liu, Jian-Gang Ren, Wei Zhang, Xin-Xin Wang, Zi-Li Yu, Qiu-Yun Fu, Xue-Peng Xiong, Jun Jia, Bing Liu, and Gang Chen

Table S1. Clinical characteristics and response outcomes of OSCC patients undergoing immunotherapy. Related to Figure 1.

Patient ID	Gender	Age	Grade	Stage	Treatment	Pathologic response (tumor regression rate)	
1	Male	52	IVA	T4aN0M0	Im	pNR	0.00%
2	Male	56	III	T3N0M0	ImC	MPR	99.00%
3	Male	33	III	T2N1M0	Im	pNR	0.00%
4	Male	53	IVA	T3N2bM0	Im	MPR	93.00%
5	Female	63	IVA	T4aN0M0	Im	MPR	91.70%
6	Female	50	III	T3N0M0	ImC	pPR	62.55%
7	Male	52	IVA	T4aN2bM0	ImC	MPR	91.30%
8	Male	61	IVA	T3N2bM0	Im	pPR	67.13%
9	Male	49	IVA	T4aN0M0	ImC	MPR	97.89%
10	Male	48	IVA	T4aN2cM0	ImC	MPR	99.00%
11	Male	62	III	T3N0M0	Im	pNR	22.00%
12	Male	44	III	T3N0M0	Im	pNR	0.00%
13	Male	37	III	T3N0M0	Im	pPR	84.17%
14	Male	48	IVA	T3N2cM0	ImC	pPR	72.00%
15	Male	64	IVA	T4aN0M0	Im	pNR	0.00%
16	Male	63	III	T3N0M0	Im	pNR	33.00%
17	Male	67	III	T2N1M0	Im	MPR	99.00%
18	Male	65	III	T3N0M0	ImC	pCR	100.00%
19	Female	55	IVA	T4aN0M0	Im	pNR	0.00%
20	Male	39	IVA	T4aN1M0	ImC	MPR	97.49%
21	Male	52	IVA	T2N2bM0	ImC	MPR	99.00%
22	Female	66	III	T3N0M0	Im	pNR	28.16%
23	Male	65	III	T3N0M0	ImC	pPR	70.31%
24	Male	67	IVA	T4aN0M0	ImC	MPR	97.67%
25	Male	35	III	T3N0M0	ImC	MPR	97.41%
26	Male	33	IVA	T3N2cM0	ImC	MPR	99.00%
27	Female	64	III	T3N0M0	Im	pNR	0.00%
28	Male	50	IVA	T4aN0M0	Im	pNR	0.00%
29	Male	45	IVA	T3N2cM0	ImC	MPR	99.00%
30	Male	63	IVA	T4aN0M0	ImC	pCR	100.00%
31	Male	50	III	T3N0M0	ImC	pCR	100.00%
32	Male	34	IVA	T4aN0M0	Im	pNR	0.00%
33	Male	52	III	T3N0M0	Im	pPR	57.73%
34	Female	34	IVA	T3N2bM0	ImC	Non-evaluable	Non-evaluable
35	Male	65	IVA	T4aN0M0	Im	pNR	7.55%
36	Male	47	III	T2N1M0	ImC	MPR	90.00%
37	Male	65	III	T2N1M0	Im	pNR	0.00%
38	Male	42	III	T3N1M0	ImC	pCR	100.00%

39	Male	62	IVA	T4aN1M0	ImC	pPR	72.39%
40	Male	46	III	T3N0M0	Im	pNR	0.00%
41	Male	37	IVA	T2N2bM0	ImC	pNR	0.00%
42	Male	44	III	T3N0M0	ImC	MPR	99.00%
43	Male	38	III	T3N1M0	ImC	MPR	99.00%
44	Male	33	III	T3N1M0	Im	pNR	14.52%
45	Male	32	III	T3N0M0	Im	pPR	85.00%
46	Male	47	III	T3N0M0	Im	MPR	99.00%
47	Female	59	IVA	T4aN0M0	Im	pNR	0.00%
48	Male	52	IVA	T2N2bM0	Im	pNR	0.00%
49	Male	61	IVA	T2N2bM0	Im	pNR	0.00%
50	Female	44	III	T3N0M0	Im	pNR	0.00%
51	Male	66	III	T3N0M0	Im	pNR	0.00%
52	Male	39	III	T2N1M0	ImC	MPR	99.00%
53	Female	53	IVA	T2N2cM0	ImC	pCR	100.00%
54	Male	66	IVA	T2N2aM0	ImC	MPR	99.00%
55	Male	49	III	T3N1M0	ImC	Non-evaluable	Non-evaluable
56	Male	50	III	T3N1M0	ImC	pNR	0.00%
57	Male	52	IVA	T4aN2bM0	Im	pNR	0.00%
58	Male	36	III	T3N0M0	Im	pNR	0.00%
59	Male	66	III	T3N1M0	Im	pNR	32.33%
60	Male	50	III	T3N0M0	ImC	pCR	100.00%
61	Male	68	III	T3N0M0	ImC	pCR	100.00%
62	Male	46	III	T3N0M0	ImC	pCR	100.00%
63	Male	58	III	T2N1M0	ImC	pCR	100.00%
64	Male	36	IVA	T2N2aM0	ImC	MPR	99.00%
65	Male	42	III	T3N0M0	Im	pNR	0.00%
66	Male	48	III	T2N1M0	ImC	pCR	100.00%
67	Male	39	III	T3N0M0	Im	pNR	43.10%
68	Male	43	III	T2N1M0	Im	MPR	97.14%

Abbreviations: ImC, Immunochemotherapy; Im, Immunotherapy; pCR, pathologic complete response, MPR, major pathologic response; pPR, pathologic partial response; pNR, pathologic non-response.

Table S2. Summary of quality control (QC) metrics for individual patient samples. Related to Figure 2.

Patient ID	UMIs (median)	Genes	Genes (median)	Cell
10pre	8743	22603	2508	5606
10post	10626	23078	2560	2589
13pre	4903	23676	1635	5331
13post	4388	22537	1301	4842
14pre	6377	23861	2021	5366
14post	4341	23874	1472	8832
15pre	10368	25773	2873	6986
15post	4781	23506	1723	6735
16pre	6689	24008	2034	5186
16post	8662	25076	2617	5371
17pre	5935	24102	1588	3373
17post	4014	23255	1365	4115
18pre	6620	24584	2376	6889
18post	4944	22811	1625	3949
19pre	8256	25235	2538	9527
19post	6391	22471	2054	5074
22pre	2479	22020	901	5299
22post	4067	23130	1428	6958
27pre	4512	23329	1746	8085
27post	6337	21774	2124	3074
35pre	7494	25554	2484	8376
35post	4613	24345	1821	8352
37pre	4784	25173	1751	7882
37post	4133	25787	1460	7756
40pre	6717	24285	1760	7709
40post	9555	24550	2464	5038
49post	10909	24243	2813	6175
59post	4653	22148	1582	5223
62post	4349	22229	1425	6379
63post	4098	23523	1461	5568
64pre	12671	23410	2921	7049
64post	3463	22648	1288	4742
65pre	6793	23173	2124	3404
65post	9060	24278	2531	5072
66pre	2761	23203	1142	6363
66post	7020	23335	1840	5825
67pre	3142	21410	1217	4157
67post	3938	23093	1302	6763
68pre	5207	22279	1772	4220
68post	3948	21478	1362	5217

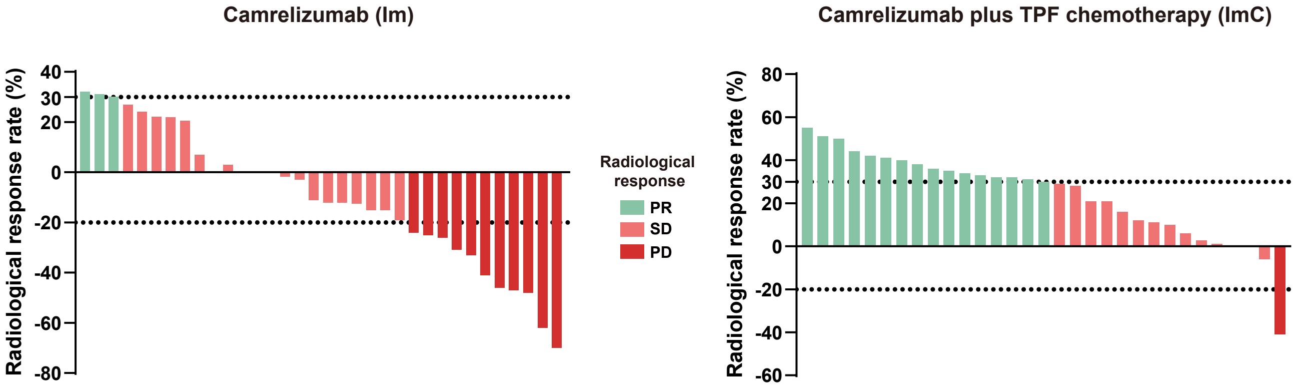

Figure S1. Waterfall plot showing patients' radiological response in arm Im and ImC. Related to Figure 1.

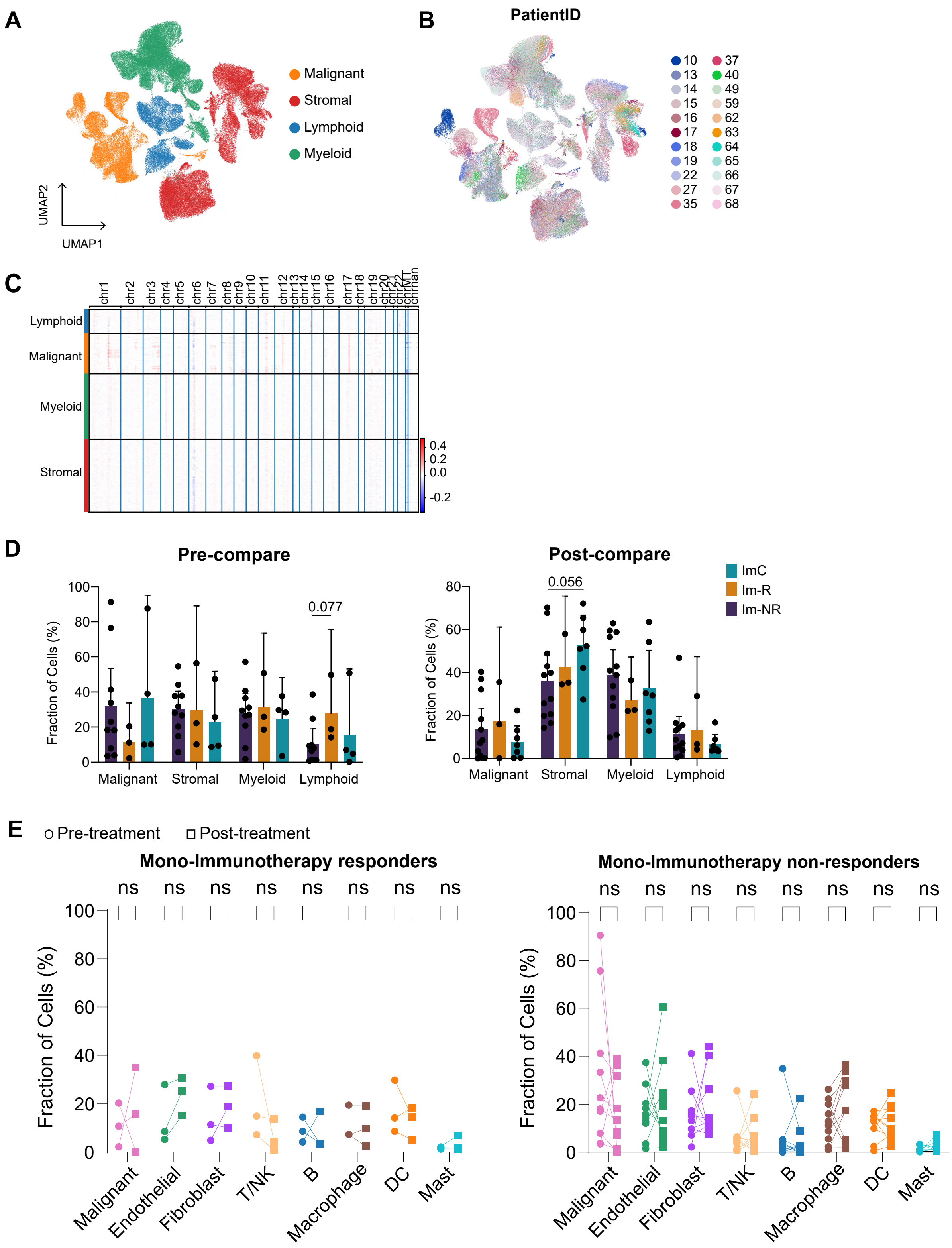

Figure S2. Comparison of cell type distribution before and after neoadjuvant immunotherapy in OSCC patients. Related to Figure 2. (A and B) UMAP plot categorizes all cells into malignant, stromal, lymphoid, myeloid types and showing the integration of all samples across patients in all clusters. (C) Heatmap depicting copy number variation (CNV) levels across different cell types in OSCC patients. (D) Stacked bar chart detailing the cell type composition for each patient pre- and post-immunotherapy treatment, grouped by response categories: ImC (Immunochemotherapy), Im-R (Immunotherapy response), and Im-NR (Immunotherapy non-response). Data is represented as mean ± SD. (E) Line plots illustrating the change in cellular fractions in paired samples pre- and post-immunotherapy treatment. The Wilcoxon rank-sum test tests statistical significance. *, $P < 0.05$; **, $P < 0.01$, ns, not significant.

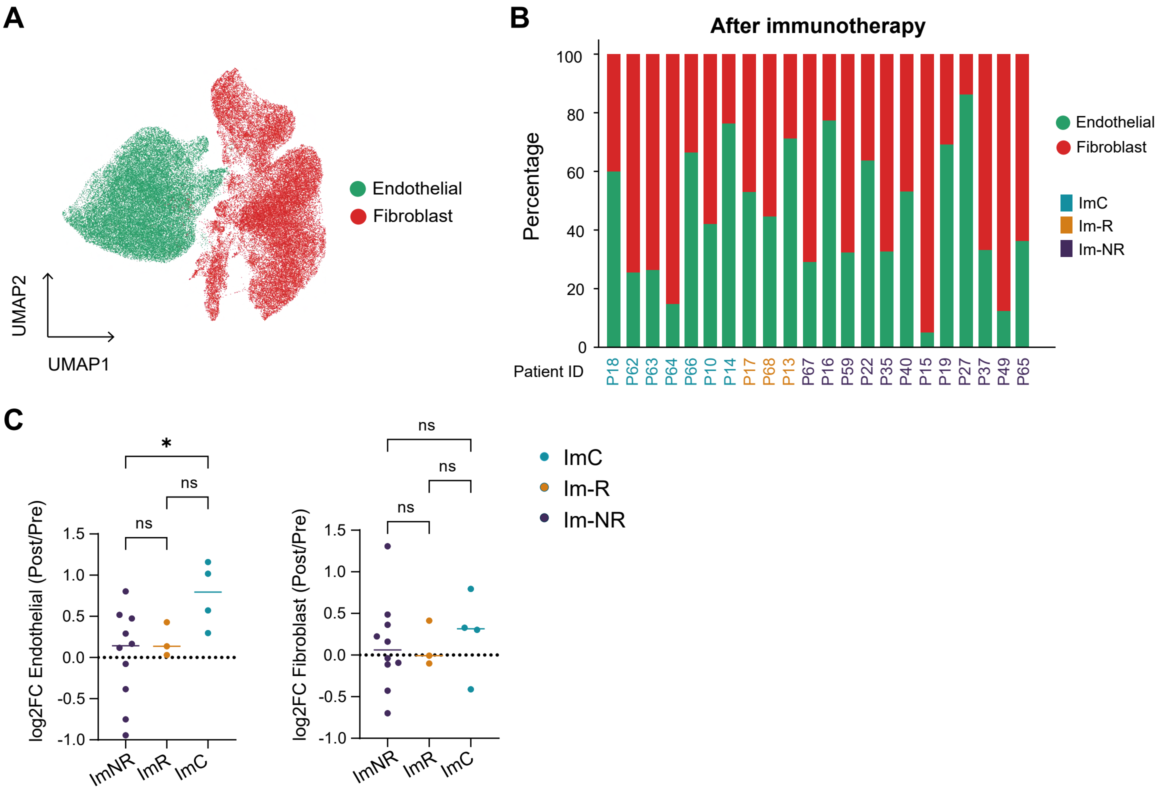

Figure S3. Comparison of post-immunotherapy stromal cell subsets in OSCC patients. Related to Figure 2.
(A) UMAP plot of stromal cells (n=81164) segregated by endothelial and fibroblast subsets. (B) Stacked bar chart illustrating the post-immunotherapy composition of endothelial and fibroblast cells across patients, with immunotherapy groups categorized as ImC (Immunochemotherapy), Im-R (Immunotherapy response), and Im-NR (Immunotherapy non-response). (C) Graphs showing log2 fold changes in endothelial and fibroblast subsets, grouped by immunotherapy response category. Horizonal bars indicate median. *, $P < 0.05$, ns, not significant.

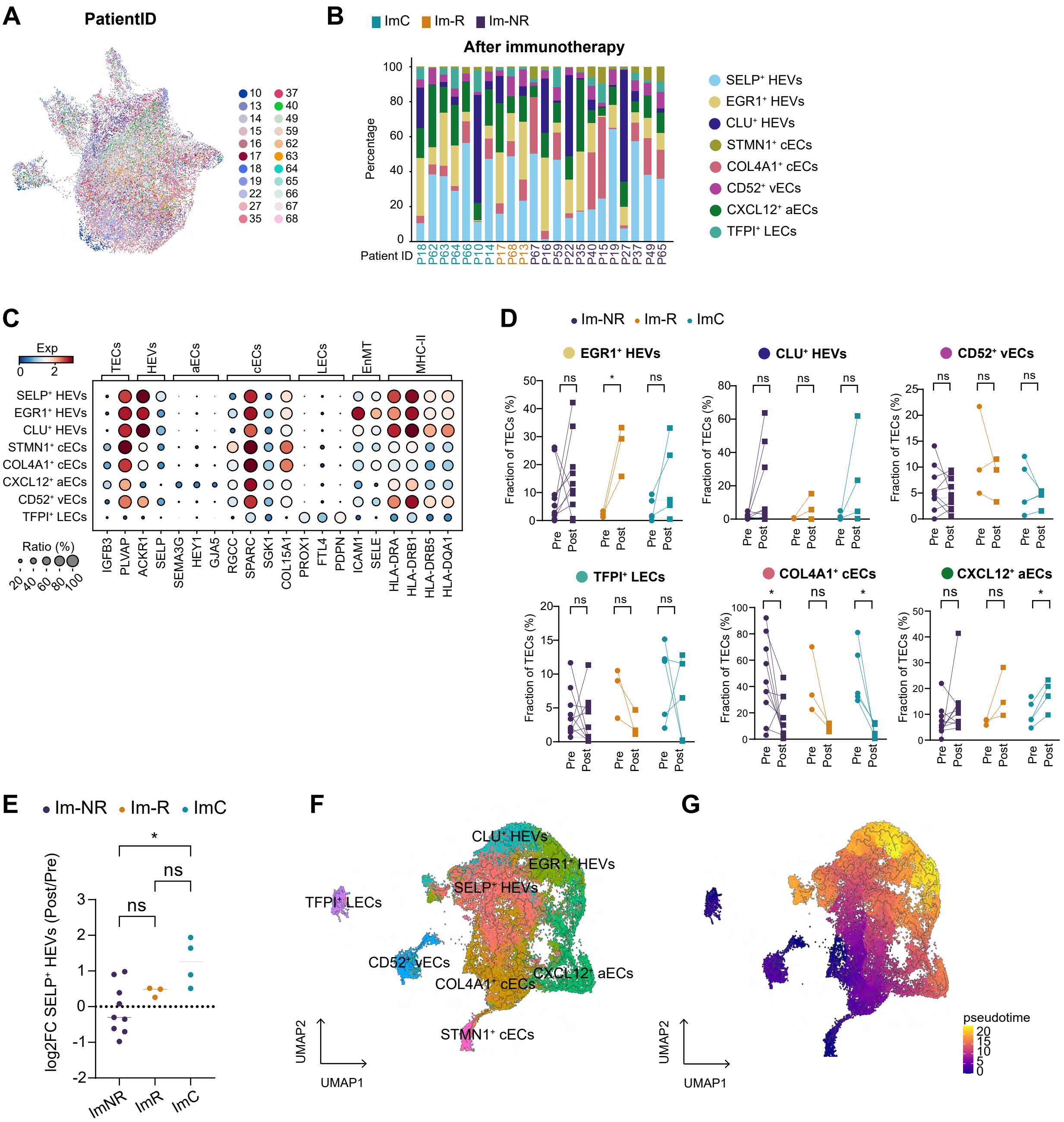

Figure S4. Endothelial cell subsets and dynamics before and after neoadjuvant immunotherapy in OSCC patients. Related to Figure 3. (A) UMAP plot showing the integration of all samples across patients in endothelial clusters. (B) The stacked bar chart depicting the post-immunotherapy distribution of endothelial cell subpopulations in OSCC patients, with immunotherapy groups categorized as ImC (Immunochemotherapy), Im-R (Immunotherapy response), and Im-NR (Immunotherapy non-response). (C) Dot plots showing the expression of signature genes across endothelial subtypes, with dot size and color intensity representing expression proportion and level, respectively. (D) Paired analysis graphs detailing the fraction of tumor endothelial cells (TECs) for specific subgroups ($EGR1^+$, CLU^+, $CD52^+$, $TFPI^+$, $COL4A1^+$, $CXCL12^+$) during immunotherapy. (E) Scatter plot showing log2 fold changes of $SELP^+$ HEVs in TME after immunotherapy grouped by treatment response. Horizonal bars indicate median. (F) UMAP plot color-coded by cell subtype revealing trajectory distribution. (G) UMAP projection displaying the progression of endothelial cell states over pseudotime, with colors transitioning from cold to warm to illustrate the developmental trajectory post-treatment. The Wilcoxon rank-sum test shows statistical significance. *, $P < 0.05$; **, $P < 0.01$; ns, not significant.

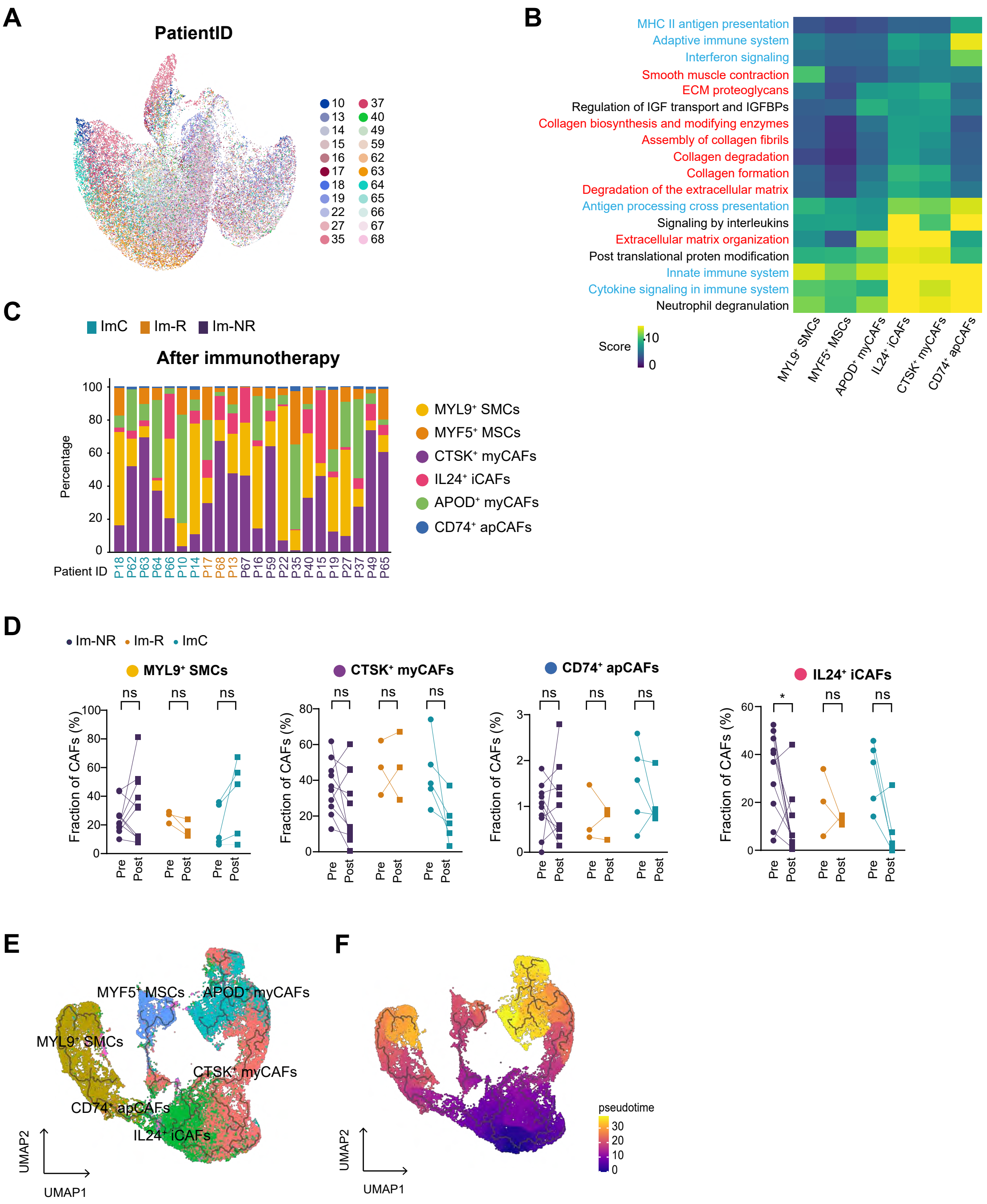

Figure S5. Fibroblast subsets and dynamics before and after neoadjuvant immunotherapy in OSCC patients. Related to Figure 4. (A) UMAP plot showing the integration of all samples across patients in fibroblast clusters. (B) Heatmap illustrating gene ontology enrichment scores for processes including antigen presentation and collagen organization. (C) A stacked bar chart illustrating the percentage of post-immunotherapy fibroblast subpopulations in OSCC patients, with immunotherapy groups categorized as ImC (Immunochemotherapy), Im-R (Immunotherapy response), and Im-NR (Immunotherapy non-response). (D) Line graphs displaying the change in the fraction of specific fibroblast subtypes ($MYL9^+$, $CTSK^+$, $CD74^+$, $IL24^+$) before (Pre) and after (Post) immunotherapy, with 'ns' indicating non-significant differences. (E) A UMAP plot delineating the distribution of fibroblast subtypes within the tumor microenvironment. (F) A UMAP projection depicting the developmental trajectory of fibroblast subtypes over pseudotime after immunotherapy, with the color gradient representing progression from initial to final states. The Wilcoxon rank-sum test shows statistical significance. *, $P < 0.05$; **, $P < 0.01$; ns, not significant.

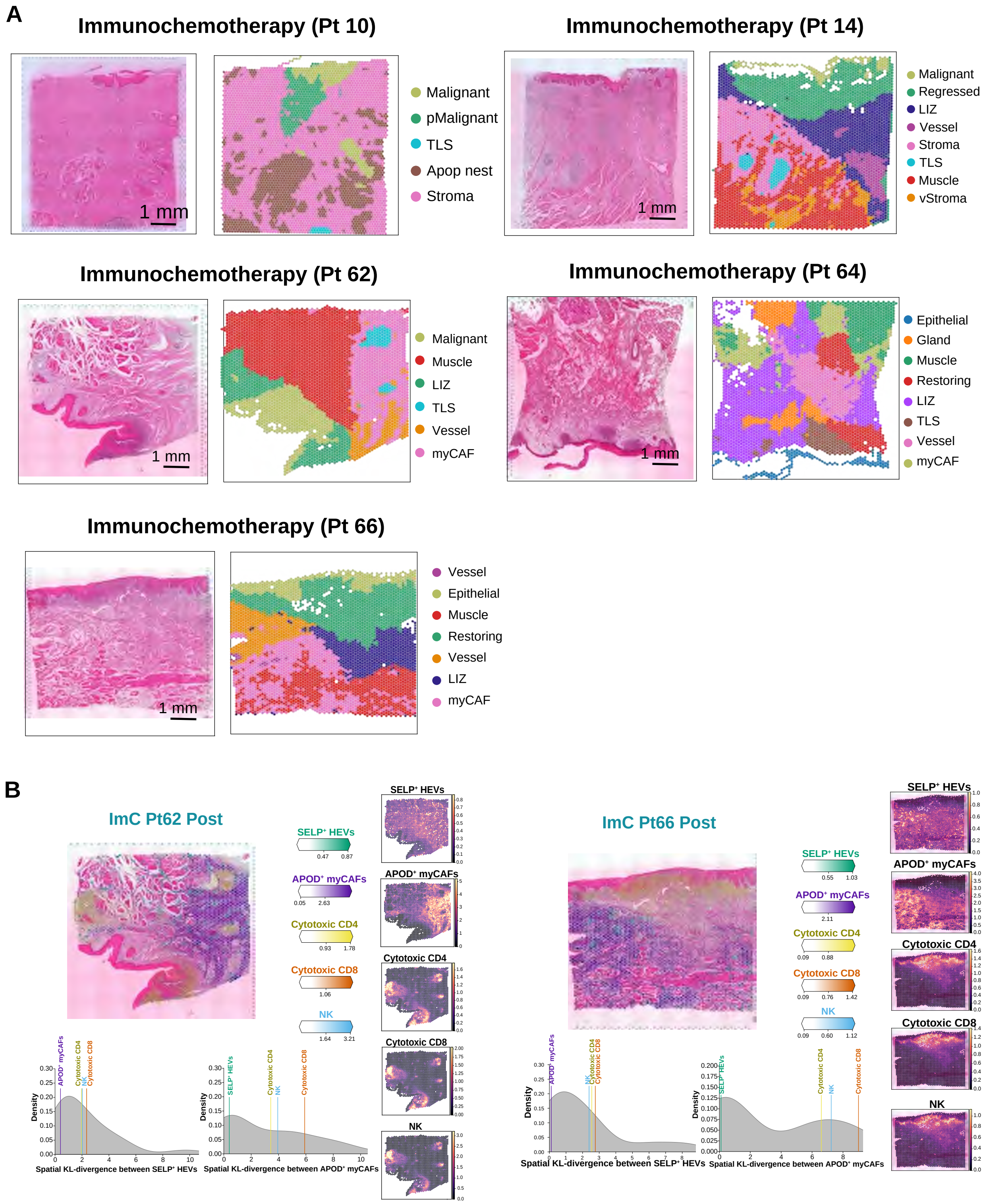

Figure S6. Overall landscapes of spatial transcriptomics in OSCC patients after immunochemotherapy. Related to Figure 5.
(A) Post immunochemotherapy (Patient #10, #14, #62, #64, #66) presenting a comparative view of histological tissue structure and a spatial transcriptomic map, identifying key cellular zones including epithelial region, malignant region, partial malignant region, regression region, restoring region, lymphoid cells infiltration zones (LIZ), vessels, stromal region, tertiary lymphoid structures (TLS), apoptosis nest, myCAF, gland, muscle fibers and vessels infiltration stroma (vStroma). Scale bar, 1 mm. (B) Estimated cell abundance is depicted by color intensity for interesting cell types (top left) as well as individually for each cell type (top right). The density plots illustrate the Kullback-Leibler (KL) divergence comparing the observed values between two clusters against the null distribution (bottom).

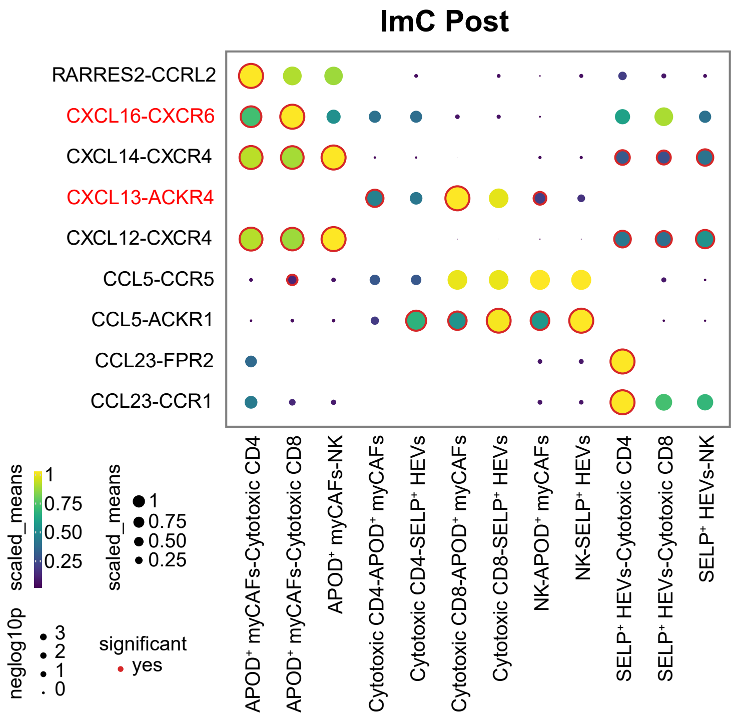

Figure S7. CellPhoneDB analysis of ligand-receptor of chemokine across cytotoxic cells and immunochemotherapy-friendly stromal cells in post-immunochemotherapy. Related to Figure 5.

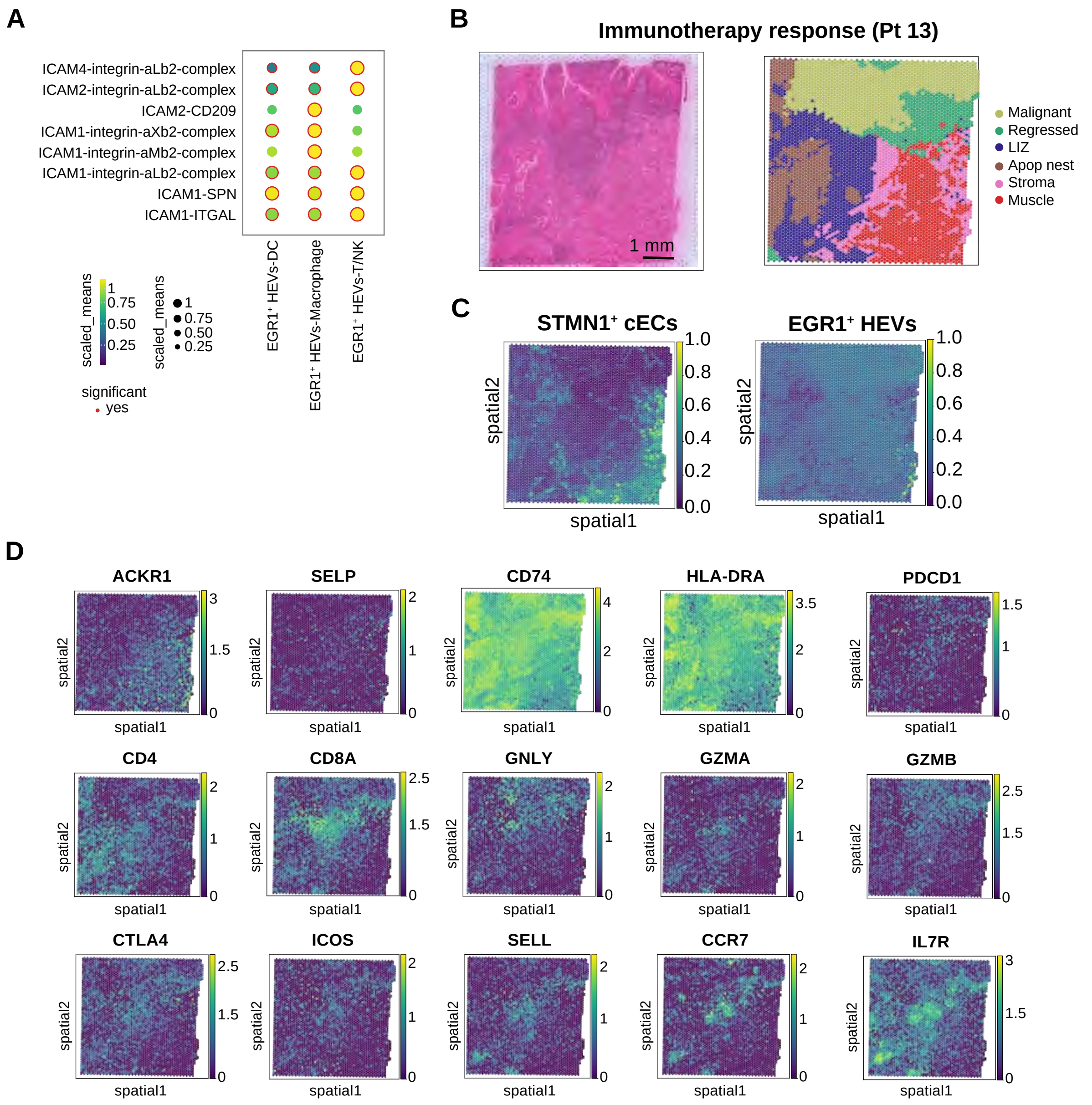

Figure S8. Spatial distribution of TEC subpopulations and T/NK markers in OSCC mono-immunotherapy responder. Related to Figure 5. (A) Dot plot matrix highlighting ICAM signaling interactions between EGR1^{+} HEVs and immune cells during immunotherapy. (B) Post immunochemotherapy (Patient #13) presenting a comparative view of histological tissue structure and a spatial transcriptomic map, identifying key cellular zones including malignant region, regression region, lymphoid cells infiltration zones (LIZ), apoptosis nest, stromal region, muscle. Scale bar, 1 mm. (C) Spatial heatmaps showing abundant STMN1^{+} cECs and EGR1^{+} HEVs in tumor regression regions. (D) The distribution of immune cell markers such as *ACKR1*, *CD4*, *CD8A*, cytotoxicity and antigen-presenting molecules were shown in tumor regression zones.

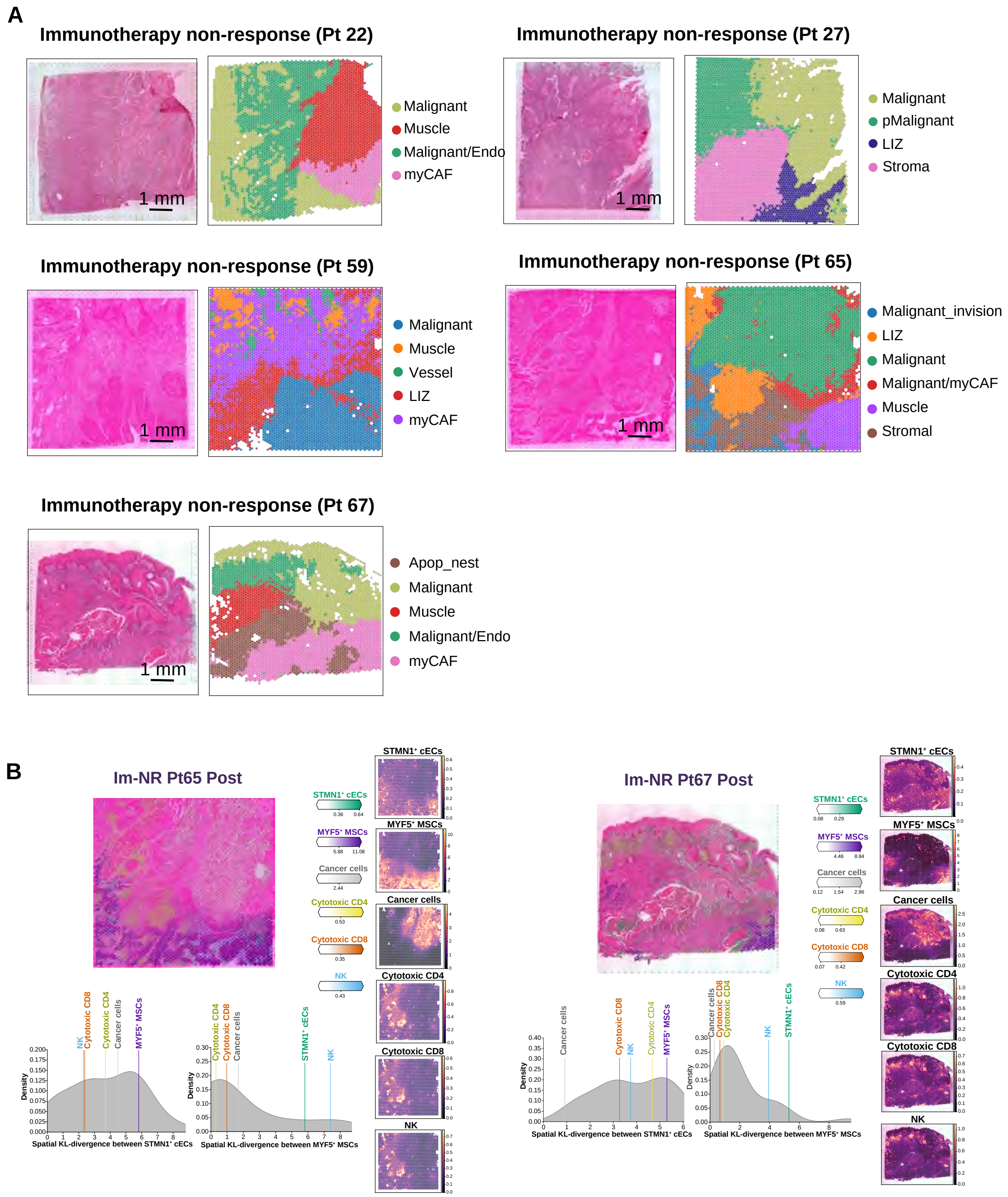

Figure S9. Overall landscapes of spatial transcriptomics in OSCC patients after immunotherapy. Related to Figure 6.
(A) Post immunotherapy non-responders (Patient #22, #27, #59, #65, #67) presenting a comparative view of histological tissue structure and a spatial transcriptomic map, identifying key cellular zones including malignant region, partial malignant region, apoptosis nest, lymphoid cells infiltration zones (LIZ), vessels, stromal region, muscle fibers and myCAF. Scale bar, 1 mm. (B) Estimated cell abundance is depicted by color intensity for interesting cell types (top left) as well as individually for each cell type (top right). The density plots illustrate the Kullback-Leibler (KL) divergence comparing the observed values between two clusters against the null distribution (bottom).

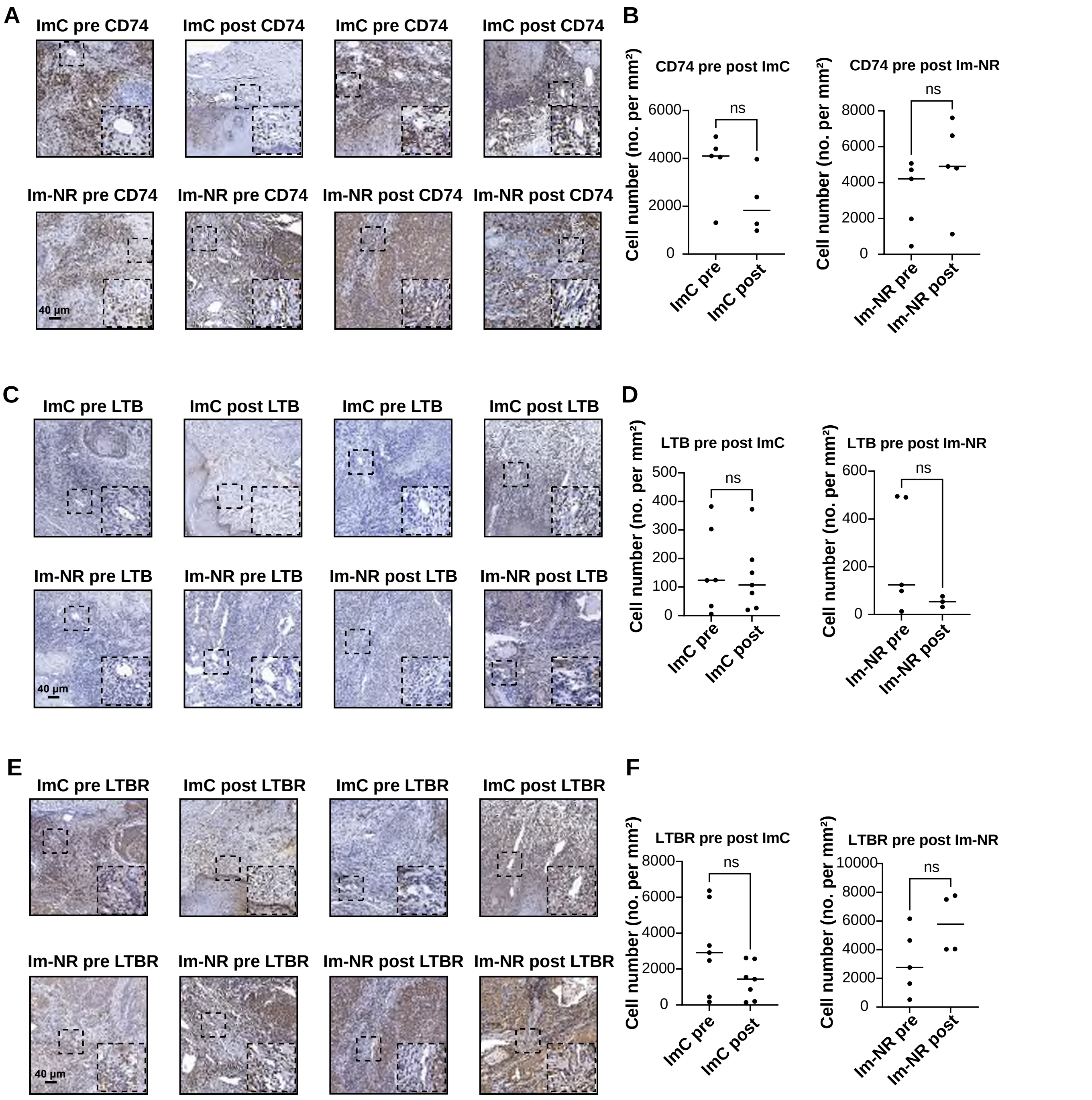

Figure S10. Immunohistochemical staining of LTB, LTBR and CD74 in OSCC patients undergoing immunotherapy and immunochemotherapy. Related to Figure 7.

(A) Immunohistochemical staining of pre-therapy and post-therapy CD74 in OSCC patients undergoing immunotherapy and immunochemotherapy. Scale bar, 40 µm. (B) Histogram of quantification of pre-therapy and post-therapy CD74 in OSCC patients undergoing immunotherapy and immunochemotherapy. Horizonal bars indicate median. (C) Immunohistochemical staining of pre-therapy and post-therapy LTB in OSCC patients undergoing immunotherapy and immunochemotherapy. Scale bar, 40 µm. (D) Histogram of quantification of pre-therapy and post-therapy LTB in OSCC patients undergoing immunotherapy and immunochemotherapy. Horizonal bars indicate median. (E) Immunohistochemical staining of pre-therapy and post-therapy LTBR in OSCC patients undergoing immunotherapy and immunochemotherapy. Scale bar, 40 µm. (F) Histogram of quantification of pre-therapy and post-therapy LTBR in OSCC patients undergoing immunotherapy and immunochemotherapy.
Horizonal bars indicate median.
