# Supplementary material for: Immune-featured stromal niches associate with response to neoadjuvant immunotherapy in oral squamous cell carcinoma
Source: Cell Rep Med. 2025 Mar 18;6(3):102024. doi: 10.1016/j.xcrm.2025.102024 (PMC11970382; doi:10.1016/j.xcrm.2025.102024)
Supplement: Document S2. Article plus supplemental information [file mmc2.pdf]

Cell Reports
Medicine

Article

Immune-featured stromal niches associate with response to neoadjuvant immunotherapy in oral squamous cell carcinoma

Graphical abstract

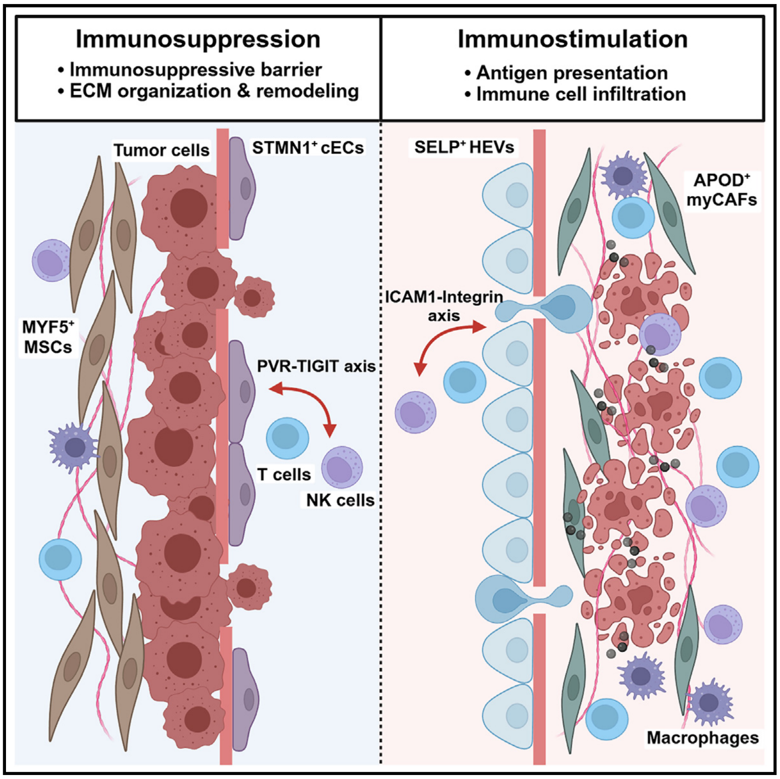

Highlights

- Two distinct stromal niches influence immunotherapy response in patients with OSCC
- SELP^{+} HEVs and APOD^{+} myCAFs enhance immune infiltration and antigen presentation
- STMN1^{+} cECs and MYF5^{+} MSCs suppress T/NK cells in the tumor microenvironment

Authors

Yu-Tong Liu, Hai-Ming Liu, Jian-Gang Ren, ..., Jun Jia, Bing Liu, Gang Chen

Correspondence

geraldchan@whu.edu.cn

In brief

Liu et al. reveal distinct tumor stromal niches in oral squamous cell carcinoma using single-cell and spatial transcriptomics. They identify SELP^{+} HEVs and APOD^{+} myCAFs as immunomodulatory cell types enhancing responses to immunotherapy. In contrast, STMN1^{+} cECs and MYF5^{+} MSCs are suppressive cell types inhibiting immune reactions.

Liu et al., 2025, Cell Reports Medicine *6*, 102024
March 18, 2025 © 2025 The Author(s). Published by Elsevier Inc.
https://doi.org/10.1016/j.xcrm.2025.102024

CellPress

Cell Reports Medicine

CellPress
OPEN ACCESS

Article

Immune-featured stromal niches associate with response to neoadjuvant immunotherapy in oral squamous cell carcinoma

Yu-Tong Liu,[1,5] Hai-Ming Liu,[1,5] Jian-Gang Ren,[1,2] Wei Zhang,[1,2] Xin-Xin Wang,[1] Zi-Li Yu,[1,2] Qiu-Yun Fu,[1,2] Xue-Peng Xiong,[1,2] Jun Jia,[1,2] Bing Liu,[1,2] and Gang Chen[1,2,3,4,6,*]

[1]State Key Laboratory of Oral & Maxillofacial Reconstruction and Regeneration, Key Laboratory of Oral Biomedicine Ministry of Education, Hubei Key Laboratory of Stomatology, School & Hospital of Stomatology, Wuhan University, Wuhan, China

[2]Department of Oral and Maxillofacial Surgery, School and Hospital of Stomatology, Wuhan University, Wuhan, China

[3]TaiKang Center for Life and Medical Sciences, Wuhan University, Wuhan, China

[4]Frontier Science Center for Immunology and Metabolism, Wuhan University, Wuhan, China

[5]These authors contributed equally

[6]Lead contact

*Correspondence: geraldchan@whu.edu.cn

https://doi.org/10.1016/j.xcrm.2025.102024

SUMMARY

Tumor stromal cells (TSCs) play a crucial yet underexplored role in the tumor microenvironment (TME). This study uses single-cell sequencing and spatial transcriptomics on paired tumor specimens from 22 patients with oral squamous cell carcinoma (OSCC) enrolled in a randomized two-arm phase 2 trial, receiving neoadjuvant anti-PD-1 mono-immunotherapy or anti-PD-1 plus docetaxel-cisplatin-5-fluorouracil (TPF) immunochemotherapy. Single-cell analysis reveals increased TSCs within the TME of responders in immunochemotherapy. Notably, significant post-treatment upregulation of SELP$^+$ high endothelial venules (HEVs) and APOD$^+$ myofibroblastic cancer-associated fibroblasts (myCAFs), alongside a decline in STMN1$^+$ capillary endothelial cells (cECs), is specific to the immunochemotherapy cohort. In contrast, MYF5$^+$ muscle satellite cells (MSCs) are upregulated in non-responders to mono-immunotherapy. SELP$^+$ HEVs and APOD$^+$ myCAFs foster favorable immunomodulatory stromal niches for improved outcomes, while STMN1$^+$ cECs and MYF5$^+$ MSCs form immunosuppressive niches in tumor invasion regions, highlighting therapeutic targets. The trial was registered at ClinicalTrials.gov, and the registration number is NCT04649476.

INTRODUCTION

The tumor microenvironment (TME) represents a complex ecosystem, characterized by a heterogeneous mix of resident and infiltrating immune cells, stromal cells, blood vessels, and extracellular matrix (ECM) components.[1,2] Tumor genesis and progression are governed by a series of genetic mutations, disruptions, and modifications within stromal cells and their surrounding matrix.[3] At the forefront of tumor invasion, specific stromal niches within the TME serve as crucial platforms for pro-tumorigenic function,[4–6] characterized by the increased expression of genes crucial for tumor-stroma communication. These niches facilitate an environment in which precancerous cells can thrive, proliferate, metastasize, and evade immunological surveillance. Thus, targeting tumor stromal cells (TSCs) is a compelling therapeutic approach for the treatment of malignant tumors,[7] underlining the significance of understanding and manipulating these stromal interactions for cancer therapy.

TSCs predominantly consist of tumor endothelial cells (TECs) and cancer-associated fibroblasts (CAFs). TECs are crucial for creating hyperpermeable blood vessels that not only nurture tumor growth but also forge routes for metastasis. On the other hand, CAFs significantly influence the behavior of tumor cells and are key mediators of immune suppression within the TME, directly affecting the effectiveness of therapeutic interventions. Although anti-angiogenic therapies (AATs) have been foundational in cancer treatment,[8] their benefits are often short-lived, leading to challenges such as inconsistent efficacy and resistance among different cancer types.[9] While targeting CAFs has shown potential in preclinical research, the findings have not been consistently translated into clinical success.[10] It is essential to acknowledge the substantial heterogeneity of both TECs and CAFs within the TME, which varies according to the tumor type and spatial location. Moreover, anti-tumor treatments can induce adaptive changes in the subtypes and functions of TECs and CAFs. A deeper exploration of the heterogeneity of TSCs, particularly their spatial-temporal dynamics during anti-tumor therapies, is essential for advancing our understanding of TSCs and for improving the outcomes of cancer therapies.

Historically, our understanding into TSCs has been primarily based on mouse models and histological analyses. Although these approaches have laid the groundwork, they often fail to

This is an open access article under the CC BY license (http://creativecommons.org/licenses/by/4.0/).

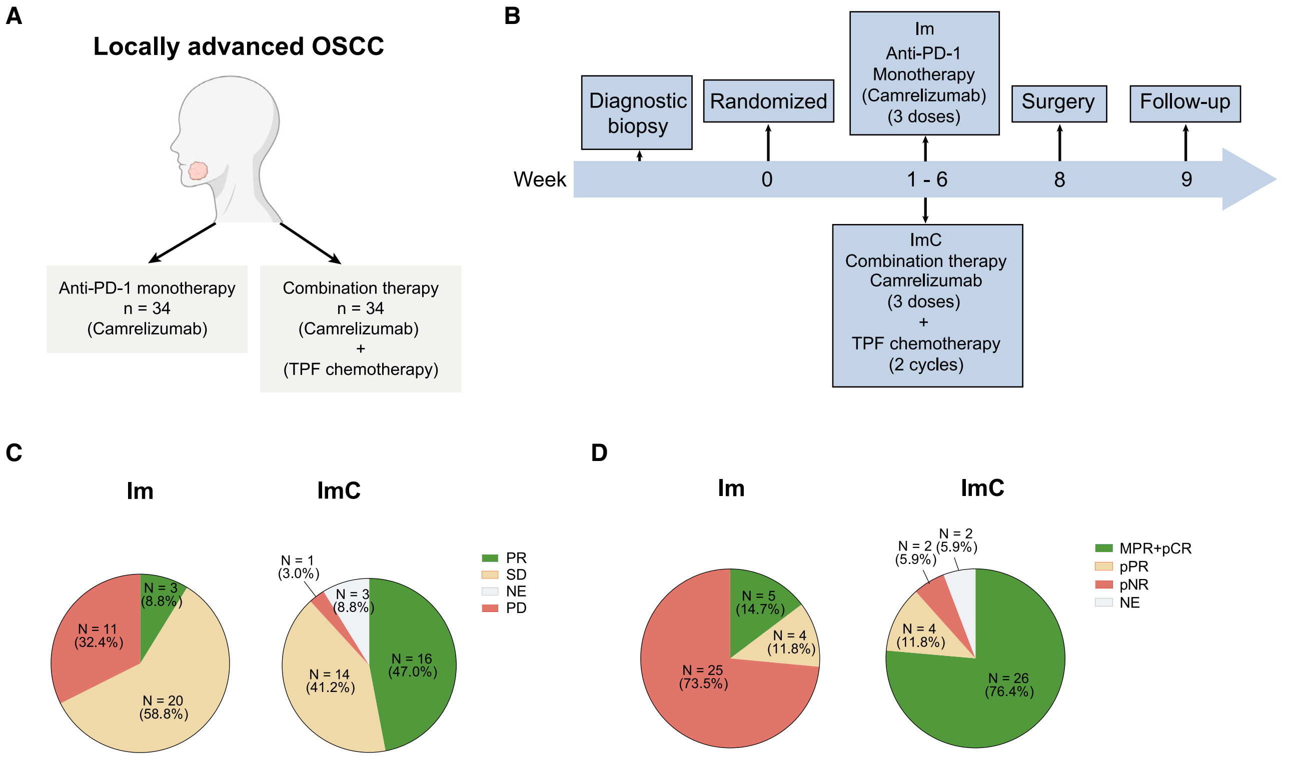

Figure 1. Trial design and patients' tumor response in the subgroups

(A) A total of 68 patients were enrolled in this trial, 34 patients were randomized to arm Im, and 34 patients were randomized to arm ImC. Im, mono-immunotherapy; ImC, immunotherapy combined with TPF chemotherapy.

(B) The two-arm design of the clinical trial. Eligible patients were treated with 3 doses of neoadjuvant camrelizumab in arm Im and 3 doses of neoadjuvant camrelizumab plus 2 cycles of TPF chemotherapy in arm ImC.

(C) Proportion of patients with radiological response in the subgroups. PR, partial response; SD, stable disease; PD, progressive disease; NE, non-evaluable.

(D) Proportion of patients with pathological response in the subgroups. pCR, pathological complete response; MPR, major pathological response; pPR, pathological partial response; pNR, pathological non-response; NE, non-evaluable.

capture the dynamic interactions and specific functions of stromal subsets within clinical contexts. The advent of advanced transcriptomic methodologies, such as RNA sequencing (RNA-seq) and single-cell RNA sequencing (scRNA-seq), has significantly enhanced our comprehension of the complexity within TSC populations.[11,12] The procurement of samples directly from individuals enrolled in clinical trials, particularly those undergoing neoadjuvant therapies, provides invaluable insights into the real-time evolution of TSCs under immune therapeutic pressure. The evaluation of TSCs, particularly those from cancers receiving immunotherapy, has garnered significant interest for its potential to identify critical stromal cell subsets and offer new therapeutic targets to improve outcomes.[13,14] However, the relatively modest success of stand-alone neoadjuvant immunotherapy has limited our ability to fully understand the anti-tumor dynamics of the stroma during treatment. Combining immunotherapy with chemotherapy, referred to as neoadjuvant immunochemotherapy (NAICT), has been shown to enhance antigen release, stimulate T cell responses, increase the rate of patients achieving pathological complete response (pCR), and significantly prolong survival.[15–18] Recent evidence has reported higher pCR rates and improved event-free survival rates in oral squamous cell carcinoma (OSCC) patients treated with NAICT[19–21]; the detailed mechanisms underlying these outcomes remain to be elucidated.

In-depth analyses of the stromal landscape under both immunotherapy and immunochemotherapy conditions are critical, particularly to distinguish between treatment responders and non-responders. These analyses are key to enhancing our understanding of how stromal cells react to different therapeutic interventions and will significantly influence the development of future treatment strategies. Leveraging the advantages of our double-arm randomized controlled clinical trial (NCT04649476), we obtained paired tumor samples from 22 patients diagnosed with OSCC. Through single-cell sequencing and spatial transcriptomics, this study unveils the dynamic changes within TSCs and elucidates their roles throughout the course of NAICT and mono-immunotherapy.

RESULTS

Clinical activity of NAICT in OSCC patients

In our clinical trial (NCT04649476), 68 locally advanced OSCC patients were enrolled and randomized to either neoadjuvant mono-immunotherapy (Im) or immunotherapy combined with docetaxel-cisplatin-5-fluorouracil (TPF) chemotherapy (ImC) (Figure 1A). The

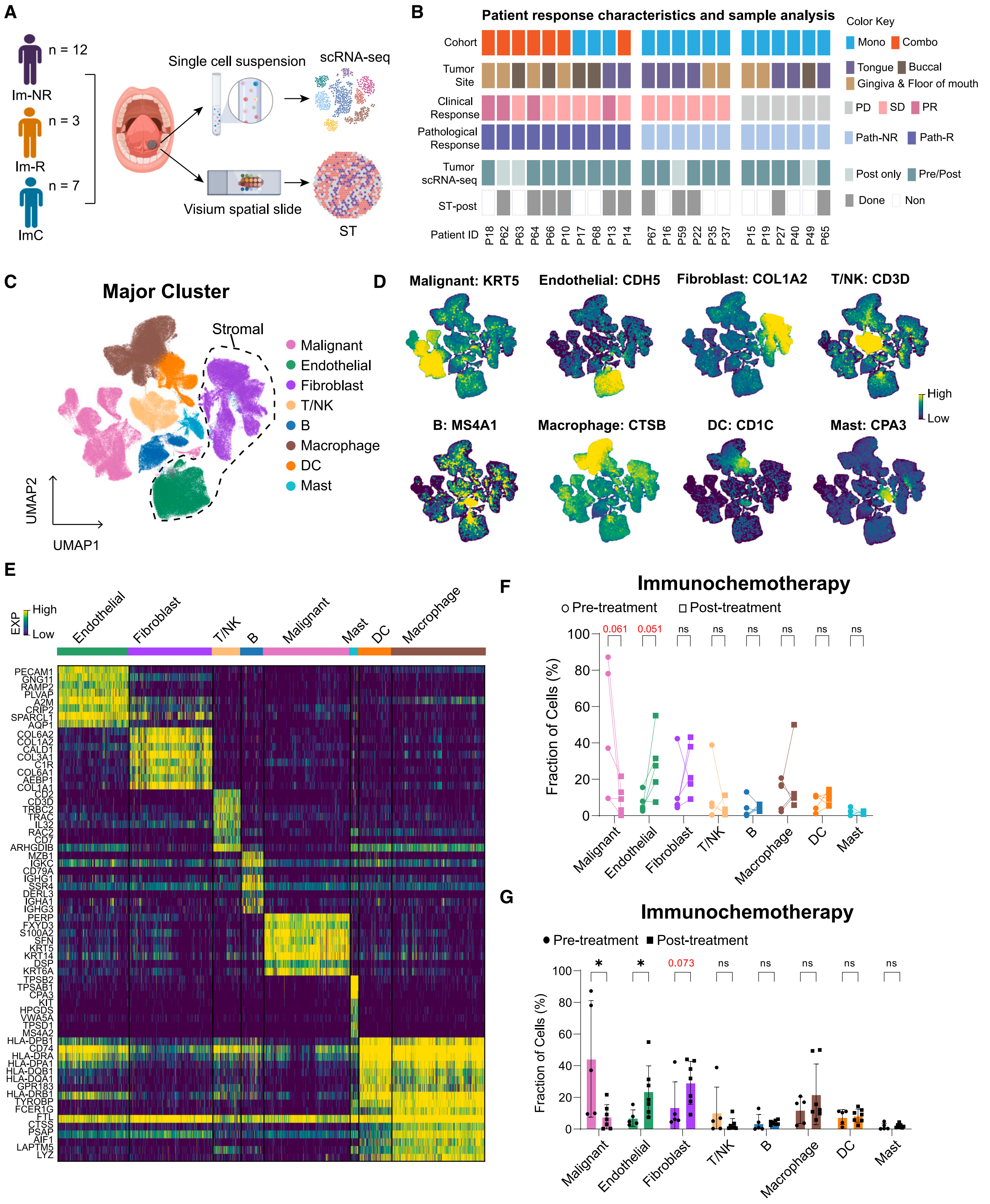

Figure 2. Comprehensive analysis of pathological responses and cellular profiles in OSCC patients undergoing immunotherapy

(A) Workflow diagram outlining the process from sample collection to scRNA-seq and spatial transcriptomic analysis.

(B) Clinical metadata and analyses performed for each patient, including response metrics and sample collection.

(legend continued on next page)

whole treatment schedule was shown in Figure 1B. In brief, patients received camrelizumab intravenously for 3 doses in arm Im and 3 doses of camrelizumab plus 2 cycles of TPF chemotherapy in arm ImC. For radiological response, 3 patients (8.8%) achieved a partial response (PR) in arm Im according to the response evaluation criteria in solid tumors (RECIST) criteria and 16 patients (47.0%) achieved PR in arm ImC (Figures 1C and S1). It was observed that 5 patients (14.7%) in arm Im and 26 patients (76.4%) in arm ImC achieved pathological major response (Figure 1D). The data showed that the addition of TPF chemotherapy to neoadjuvant immunotherapy in arm ImC significantly improved the therapeutic efficacy compared to arm Im.

Single-cell analysis reveals increased stromal cells in the TME of OSCC patients after immunochemotherapy

To gain a comprehensive understanding of the response mechanisms, particularly distinguishing responders in OSCC, 40 tumor samples from 22 patients including 12 immunotherapy non-responders, 3 immunotherapy responders, and 7 responders receiving immunochemotherapy were prepared into single-cell suspensions for scRNA-seq. Additionally, spatial transcriptomics was performed on the post-treatment tumor tissues from 5 immunotherapy non-responders, 1 immunotherapy responder, and 5 immunochemotherapy responders (Figure 2A). Detailed information on the 22 patients enrolled in this study is presented in Figure 2B and Table S1, while detailed quality control metrics for individual patient samples are provided in Table S2.

A total of 236,996 high-quality cells were categorized into four primary groups and eight major populations (malignant, endothelial, fibroblast, T/natural killer [NK], B, macrophage, dendritic, and mast cells) and visualized using graph-based uniform manifold approximation and projection (UMAP) (Figures 2C, S2A, and S2B). Malignant cells were separated based on classical epithelial malignant genes (Figure 2D) and copy-number variations (CNVs) (Figure S2C). Representative marker genes of several major clusters were visualized (Figure 2D). The differentially expressed genes across the eight major subtypes are shown in Figure 2E. High proportions of lymphoid cells (including T/NK and B cells) were observed in baseline tumor tissues from mono-immunotherapy responders, whereas stromal cells were prevalent in patients after immunochemotherapy (Figure S2D). Paired analysis revealed that an increase in the proportions of stromal cells post-treatment was observed only in immunochemotherapy patients, with no significant changes in mono-immunotherapy patients (Figures 2F and S2E). Following immunochemotherapy, there was a significant increase in endothelial and fibroblast cells, along with a decrease in malignant cells; however, no changes were noted in other cell types (Figure 2G). Additionally, our data demonstrated a marked increase in the proportion of endothelial cells in the immunochemotherapy group compared to the mono-immunotherapy groups (Figures S3A–S3C). Next, we focused on subtypes of endothelial and fibroblast cells.

Increase of SELP$^+$ HEVs as well as decrease of STMN1$^+$ cECs after immunochemotherapy

In total 37,284 endothelial cells were re-clustered into eight populations: three types of high endothelial venules (HEVs) (SELP$^+$ HEVs, EGR1$^+$ HEVs, and CLU$^+$ HEVs), two capillary endothelial cell (cEC) clusters (STMN1$^+$ cECs and COL4A1$^+$ cECs), CD52$^+$ vein endothelial cells (vECs), CXCL12$^+$ artery endothelial cells (aECs), and TFPI$^+$ lymphatic endothelial cells (LECs) (Figures 3A, 3B, S4A, and S4B). UMAP visualization highlighted several hallmark genes unique to TECs (Figure 3C). Notably, HEVs and vECs harbored high expression levels of major histocompatibility complex class II (MHC-II) genes, suggesting their potential roles in immunomodulation (Figure S4C). Subsequent Gene Ontology (GO) analysis further revealed that HEVs were more actively involved in cell adhesion and antigen processing and presentation processes than cECs (Figure 3D).

In addition, there was a significant increase in SELP$^+$ HEVs and CXCL12$^+$ aECs, as well as a decrease in STMN1$^+$ cECs and COL4A1$^+$ cECs, after immunochemotherapy, but the proportion of EGR1$^+$ HEVs, CLU$^+$ HEVs, vECs, and LECs was not significantly changed (Figure 3E). Moreover, SELP$^+$ HEVs and CXCL12$^+$ aECs showed post-treatment increases only in the immunochemotherapy group, and SELP$^+$ HEVs showed a pronouncedly increased proportion (Figures 3F, S4D, and S4E). However, post-treatment decreases in STMN1$^+$ cECs in immunotherapy responders and COL4A1$^+$ cECs in immunotherapy non-responders were detected (Figures 3F and S4D). Trajectory analysis revealed potential transformation from cECs to HEVs (Figures S4F and S4G). These data suggest that alternate SELP$^+$ HEVs, STMN1$^+$ cECs, and CXCL12$^+$ aECs may be associated with the response of OSCC patients to NAICT.

We further conducted multiplex immunohistochemistry (mIHC) on 36 tumor samples to track SELP$^+$ HEVs and STMN1$^+$ cECs in the TME before and after treatment, aiming to validate our aforementioned findings. Our analysis revealed a significant increase in the abundance of SELP$^+$ HEVs in the immunochemotherapy group compared to the mono-immunotherapy group (Figures 3G and 3H). This difference was especially pronounced post-treatment, as shown by the higher density of SELP$^+$ HEVs in immunochemotherapy samples. Furthermore, we noted an increase in STMN1$^+$ cECs post-treatment in non-responders to mono-immunotherapy. Conversely, STMN1$^+$ cECs levels decreased in responders, regardless of whether they received mono-immunotherapy or combination therapy (Figures 3I and 3J).

Post-treatment increased APOD$^+$ myCAFs in immunochemotherapy and increased MYF5$^+$ MSCs in immunotherapy non-responders

A total of 43,880 fibroblast cells were categorized into six distinct populations: two myofibroblastic CAF (myCAF) clusters (CTSK$^+$ myCAFs and APOD$^+$ myCAFs), inflammatory CAFs (iCAFs),

(C) UMAP plot categorizes all cells (n = 236,996) into malignant, endothelial, fibroblast, T/NK, B, macrophage, dendritic cell (DC), and mast cell types.
(D) UMAP plots showing representative markers for each cell type.
(E) A detailed heatmap of differential gene expression levels across cell types.
(F) Line plots illustrating the change in cellular fractions in paired samples among immunochemotherapy. Two-sided paired t test.
(G) Bar plot comparing cellular fractions pre- and post-immunochemotherapy. Data are represented as mean ± SD. Wilcoxon test; *$p < 0.05$; ns, not significant.

CellPress
OPEN ACCESS

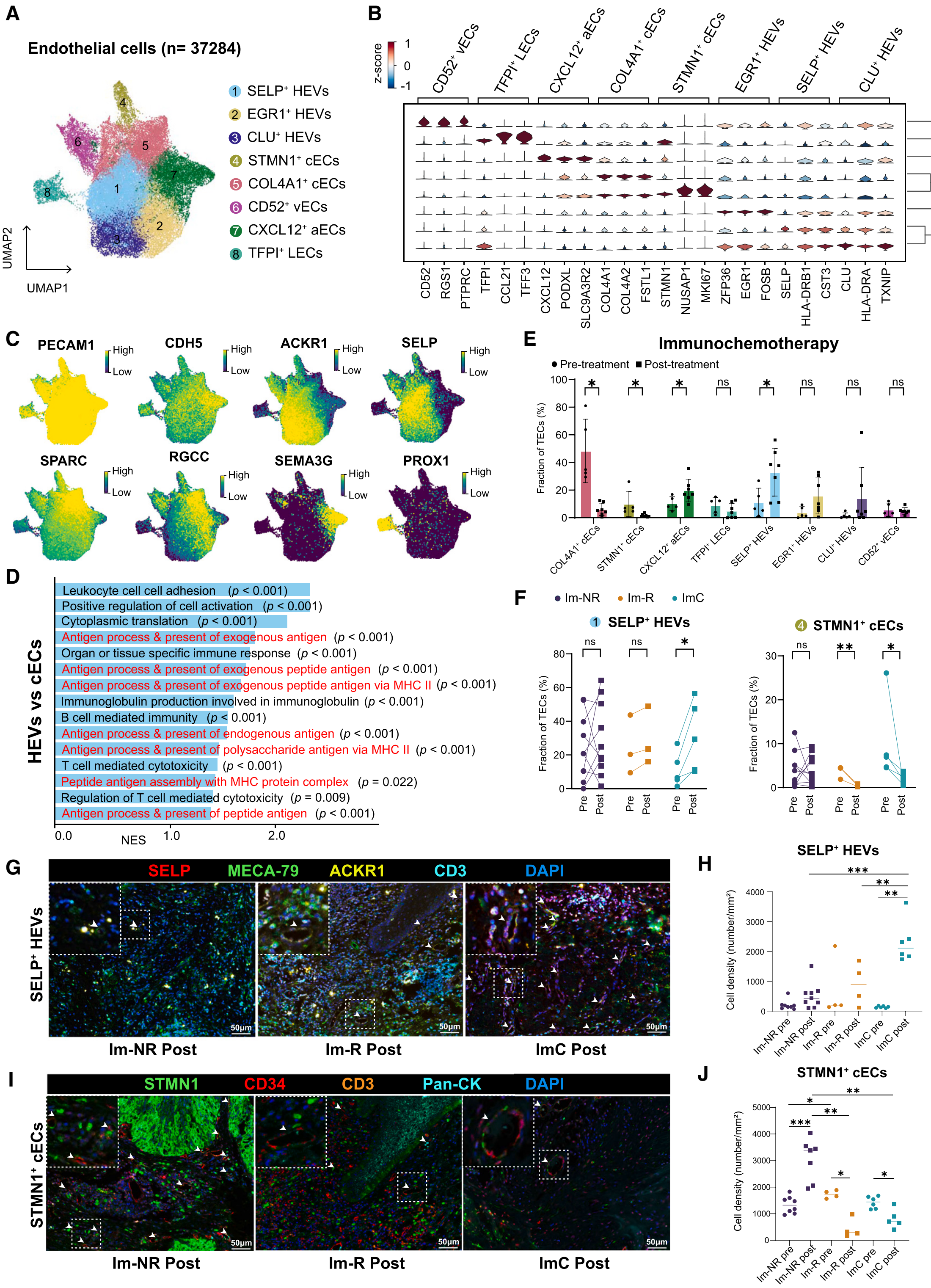

Figure 3. Characterization and dynamics of TEC subpopulations in OSCC during immunotherapy

(A) Visualization of tumor endothelial cell (TEC) subtypes (n = 37,284).

(B) Violin plots of gene expression markers for each subtype.

(C) UMAP plots indicating gene expression levels for classical TEC markers.

(legend continued on next page)

antigen-presenting CAFs (apCAFs), smooth muscle cells (SMCs), and muscle satellite cells (MSCs) (Figures 4A, 4B, and S5A). The UMAP plot also delineated a spectrum of classical markers associated with the different CAFs (Figure 4C). Notably, apCAFs were found to harbor significant expression levels of MHC-II genes, suggesting their potential roles in immunomodulation (Figure 4D). The gene set enrichment analysis data showed that myCAFs exhibited high scores for ECM remodeling, whereas apCAFs exhibited high scores for adaptive immunity (Figure S5B).

Regarding the proportion of different myCAFs, our results revealed that immunotherapy patients exhibited a higher proportion of myCAFs post-treatment, especially after immunochemotherapy (Figures 4E and S5C). Although there was a significant decrease in IL24$^+$ iCAFs after immunochemotherapy (Figure 4E), paired analysis revealed a substantial decrease in immunotherapy non-responders (Figure S5D). In contrast, post-treatment increases in APOD$^+$ myCAFs were observed only in the immunochemotherapy group (Figure 4F). Moreover, a specific and marked increase in MYF5$^+$ MSCs was detected in immunotherapy non-responders (Figure 4F). Gene set variation analysis (GSVA) analysis showed the inhibitory effects of MYF5$^+$ MSCs on classical collagen fibril organization (Figure 4G), probably resulting in a physical barrier to prevent immune cell infiltration. The proportion of other CAF subsets did not change post-treatment (Figure S5D). Trajectory analysis revealed potential transformation to APOD$^+$ myCAFs after immunochemotherapy (Figures S5E and S5F). These data suggest that alternate APOD$^+$ myCAFs and MYF5$^+$ SMCs may be associated with the NAICT response.

We subsequently performed mIHC staining for APOD$^+$ myCAFs and MYF5$^+$ MSCs to validate our findings at the protein level. At baseline, APOD$^+$ myCAFs were particularly abundant in responders to mono-immunotherapy. Post-treatment, we observed a significant increase in APOD$^+$ myCAF abundance in the immunochemotherapy group compared to the mono-immunotherapy group, underscoring the enhanced response elicited by the combination therapy (Figures 4H and 4I). Furthermore, post-treatment analysis revealed an increase in MYF5$^+$ MSCs in non-responders to mono-immunotherapy, while MYF5$^+$ MSCs levels remained consistently low in responders, regardless of whether they received mono- and combination therapies. This suggests a potential association between elevated MYF5$^+$ MSC levels and a poor therapeutic response (Figures 4J and 4K).

SELP$^+$ HEVs and APOD$^+$ myCAFs contributed to increased T/NK cell infiltration and antigen presentation after immunochemotherapy

To visualize the interaction between TSCs and immune cells within the TME and to investigate TSC function, we conducted a combined analysis of scRNA-seq and spatial transcriptomics. Notably, the analysis of cell-type abundance from the spatial transcriptome data revealed the spatial co-occurrence of stromal cells associated with immunochemotherapy. Specifically, SELP$^+$ HEVs and APOD$^+$ myCAFs were found to be co-localized in patients receiving immunochemotherapy (Figures 5A and S6). Interestingly, SELP$^+$ HEVs and APOD$^+$ myCAFs were observed to be spatially closer to cytotoxic T and NK cells than to other cell types (Figure 5A), suggesting that stromal cells favorable to immunochemotherapy are more likely co-localized with these cytotoxic immune cells.

CellPhoneDB analysis further revealed that SELP$^+$ HEVs and APOD$^+$ myCAFs exhibited strong interactions with cytotoxic T and NK cells through the ICAM1, CD55, and CD34 signaling pathways (Figure 5B). Notably, cytotoxic T and NK cells demonstrated robust engagement with SELP$^+$ HEVs via the LTA and LTB signaling pathways, which may facilitate the formation of tertiary lymphoid structures (TLSs). Additionally, APOD$^+$ myCAFs showed significant interactions with cytotoxic T and NK cells through the CXCL13 and CXCL16 signaling pathways, potentially contributing to tumor regression (Figure S7). Alternatively, EGR1$^+$ HEVs strongly interacted with immune cells via ICAM-related signaling pathways (Figures S8A–S8C). Interestingly, mono-immunotherapy responders displayed higher CD8$^+$ T cell infiltration in tumors compared to immunochemotherapy responders, potentially enabling tumor remission in these patients without TLS formation (Figure S8D).

The spatial co-localization of immunochemotherapy-favorable stromal cells and cytotoxic immune cells was further validated using mIHC staining on pre- and post-treatment samples from immunochemotherapy responders. First, we examined the spatial relationship between ICAM1$^+$SELP$^+$ HEVs and MHC-II$^+$CD3$^+$ T cells. Post-treatment, ICAM1$^+$SELP$^+$ HEVs were frequently observed at a closer distance of 120 μm from MHC-II$^+$CD3$^+$ T cells, compared to a more distant interaction of 180 μm pre-treatment (Figures 5C and 5D). This reduction in distance was accompanied by a substantial increase in the density of MHC-II$^+$CD3$^+$ T cells surrounding HEVs (Figure 5E). We also assessed the association between ICAM1$^+$APOD$^+$ myCAFs and MHC-II$^+$ T/NK cells. Post-treatment, the interaction distance between ICAM1$^+$APOD$^+$ myCAFs and MHC-II$^+$ T/NK cells decreased

(D) Bar plots depicting normalized enrichment scores for Gene Ontology terms, comparing HEVs to cECs and highlighting processes such as cell adhesion and antigen presentation.
(E) Changes in endothelial cell subtype proportions pre- and post-immunotherapy were depicted in comparative bar graphs, with statistical significance where applicable. Data are represented as mean ± SD. Wilcoxon test; *$p < 0.05$; ns, not significant.
(F) Line graphs displaying subtype percentages across patient response categories to immunotherapy or combined therapy. Two-sided paired t test; *$p < 0.05$; **$p < 0.01$; ns, not significant.
(G) Representative images of mIHC staining for SELP$^+$ HEVs in post-treatment samples. White asterisks highlight SELP$^+$ HEVs. Scale bar, 50 μm.
(H) Cell density of SELP$^+$ HEVs in baseline and post-treatment samples from mono-immunotherapy non-responders ($n = 9$), mono-immunotherapy responders ($n = 4$), and immunochemotherapy patients ($n = 6$). Horizontal bars indicate median. Two-sided unpaired t test; *$p < 0.05$; **$p < 0.01$; ***$p < 0.001$.
(I) Representative images of mIHC staining for STMN1$^+$ cECs in post-treatment samples. White asterisks highlight STMN1$^+$ cECs. Scale bar, 50 μm.
(J) Cell density of STMN1$^+$ cECs in baseline and post-treatment samples from mono-immunotherapy non-responders ($n = 8$), mono-immunotherapy responders ($n = 4$), and immunochemotherapy patients ($n = 6$). Horizontal bars indicate median. Two-sided unpaired t test.

CellPress
OPEN ACCESS

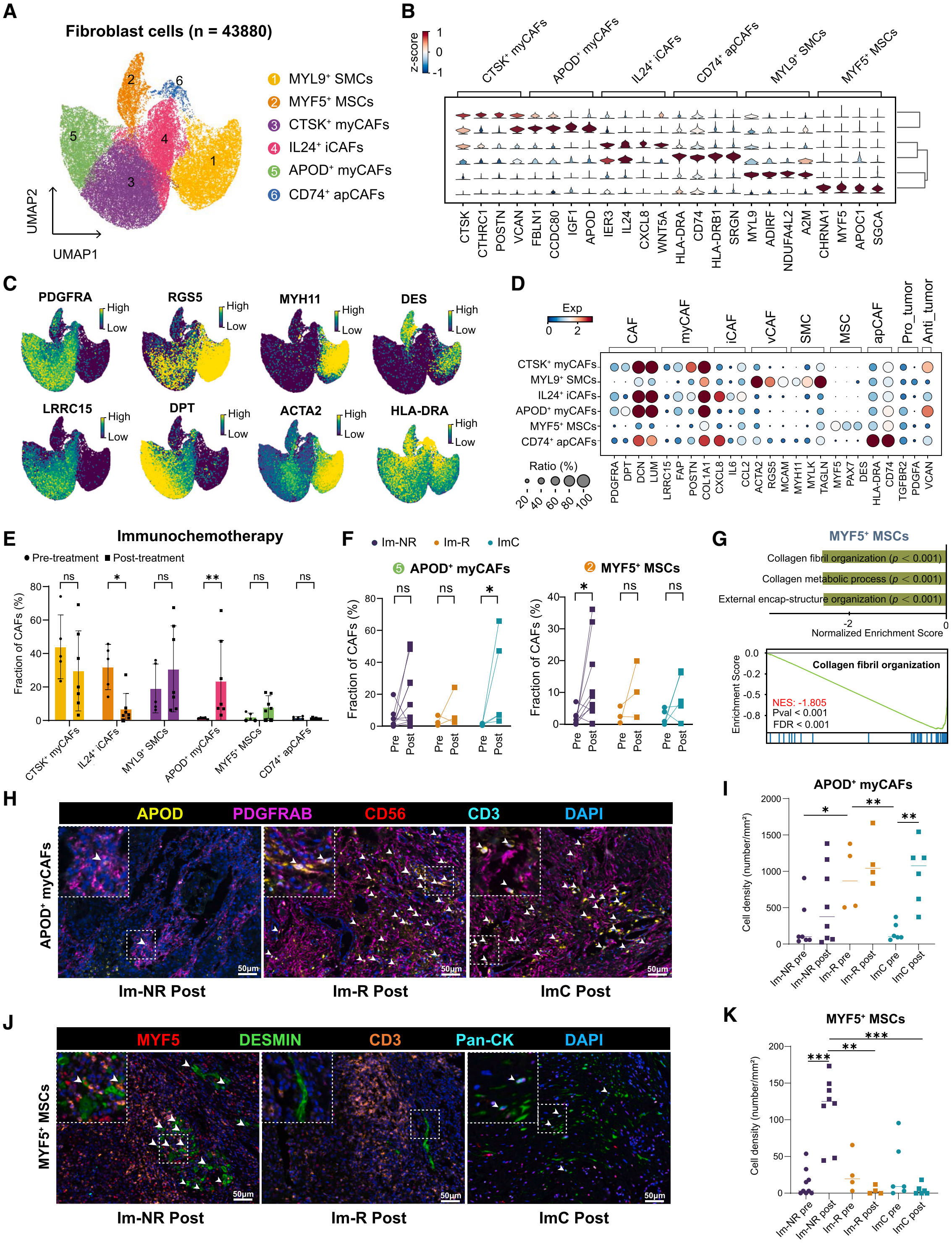

Figure 4. Characterization and dynamics of fibroblast cell subpopulations in OSCC during immunotherapy

(A) UMAP plot showing tumor-associated fibroblast cells (n = 43,880) identified in the study.

(B) Violin plots depicting differential gene expression profiles across fibroblast subtypes.

(C) UMAP plots indicating gene expression levels for classical fibroblast markers.

(D) Dot plots correlate the expression levels of fibroblast markers with dot size reflecting the proportion of cells expressing the gene and color intensity representing expression level.

(legend continued on next page)

from 180 μm pre-treatment to 120 μm (Figures 5F and 5G). Concurrently, there was a notable increase in the density of MHC-II+ T/NK cells within this interaction range surrounding the myCAFs in post-treatment samples (Figure 5H). These findings highlight that ICAM1+ stromal cells, including SELP+ HEVs and APOD+ myCAFs, play critical roles in attracting and retaining T/NK cells through ICAM1+ signaling. The increased presence of MHC-II+ T/NK cells further suggests a potential antigen-presenting capacity of ICAM1+ stromal cells, supporting a stronger immune activation response to immunochemotherapy.

STMN1+ cECs and MYF5+ MSCs enhanced immune resistance to T/NK cells in non-responders after mono-immunotherapy

A combined analysis of scRNA-seq and spatial transcriptomics revealed an abundance of STMN1+ cECs and MYF5+ MSCs at the tumor invasion front in mono-immunotherapy non-responders (Figures 6A and S9). Notably, STMN1+ cECs and MYF5+ MSCs were found to be spatially closer to cancer cells than to other cell types (Figure 6A), particularly in Pt27 and Pt59, who exhibited progressive disease. CellPhoneDB analysis indicated strong interactions between these non-responders' favorable stromal cells and cancer cells through transforming growth factor β (TGF-β) and NOTCH signaling pathways (Figure 6B). STMN1+ cECs and MYF5+ MSCs were also likely to co-localize with cytotoxic T and NK cells. In addition, they expressed immunosuppressive molecules such as PVR and NECTINs, engaging with T and NK cells via PVR/NECTIN-TIGIT signaling, which may drive adaptive immune suppression. These findings suggest that STMN1+ cECs and MYF5+ MSCs contribute to immune evasion and tumor progression by promoting interactions with cancer cells and creating an immunosuppressive microenvironment.

Using mIHC analysis, we investigated the spatial distribution and interactions between immune and stromal cells within the TME of mono-immunotherapy non-responders, both pre- and post-treatment. We observed that STMN1+ cECs frequently co-localized with CD3+ T cells, with the average interaction distance decreasing from 175 μm before treatment to 130 μm after treatment (Figures 6C and D). This closer proximity was accompanied by a notable increase in TIGIT+CD3+ T cells within this frequent interaction range in post-treatment samples, suggesting enhanced immune modulation through PVR-TIGIT signaling. Density distribution analysis further confirmed a notable clustering of PVR+CD3+ T cells around STMN1+ cECs, highlighting their role in creating a localized immune-suppressive niche (Figure 6E). Similarly, MYF5+ MSCs, identified by MYF5 and DESMIN, showed a close association with TIGIT+CD3+ T cells, with the interaction distance decreasing from 160 μm pre-treatment to 120 μm post-treatment (Figures 6F and 6G), indicating a tighter spatial association. After treatment, the density of TIGIT+CD3+ T cells surrounding MYF5+ MSCs significantly increased, reflecting a strengthened immune-suppressive interaction. Density distribution analysis corroborated these findings, showing an enriched connection of PVR+CD3+ T cells near MYF5+ MSCs in post-treatment samples (Figure 6H). Collectively, these results demonstrate that both STMN1+ cECs and MYF5+ MSCs play pivotal roles in establishing a suppressive TME by facilitating tighter spatial interactions and recruiting TIGIT+CD3+ T cells, which may substantially compromise the efficacy of immune checkpoint blockade therapy in non-responders.

SELP+ HEVs and APOD+ myCAFs create immune-favorable regions, while STMN1+ cECs and MYF5+ MSCs form immunosuppressive niches in the TME following immunotherapy

In patients undergoing immunochemotherapy, the tumor regression region displayed significant formation of TLSs and extensive immune cell infiltration (Figure S6). Notably, abundant SELP+ HEVs and APOD+ myCAFs were prominently present in these regions (Figure 7A). Spatial analysis further highlighted an enrichment of key signaling pathways, including LTB, ICAM1, CD55, and CXCL16 (Figure 7B), which are essential for facilitating T and NK cell infiltration, promoting TLS formation, and enhancing antigen processing and presentation, especially via MHC-II. However, immunohistochemistry (IHC) staining showed no significant differences in CD74 and LTB signaling between immunochemotherapy and mono-immunotherapy non-responders (Figure S10), likely due to the limited immune cell infiltration in the restoring regions. Additionally, spatial analysis indicated elevated levels of T cell-mediated cytotoxicity and antigen presentation within the tumor regression region (Figure 7C). Collectively, these findings emphasize the critical roles of SELP+ HEVs and APOD+ myCAFs in facilitating T/NK cell infiltration and enhancing antigen presentation, thereby contributing to effective tumor regression.

In non-responders to mono-immunotherapy, several key signaling pathways were identified within tumor invasion regions, which may adversely impact immune response (Figures 7D, 7E, and S10). Malignant cells exhibited strong interactions with STMN1+ cECs and MYF5+ MSCs through JAG-NOTCH pathways, with STMN1+ cECs predominantly engaging via NOTCH1 signaling and MYF5+ MSCs via NOTCH2 signaling (Figure 7E).

(E) Comparative bar graphs showing the proportion of each fibroblast subtype before and after immunotherapy. Data are represented as mean ± SD. Wilcoxon test; $*p < 0.05$; $**p < 0.01$; ns, not significant.

(F) Line graphs demonstrating the key increased subtypes post-immunotherapy in paired samples, with statistical significance indicated. Two-sided paired t test; $*p < 0.05$; ns, not significant.

(G) GSVA analysis revealing a strongly negative correlation between MYF5+ MSCs and collagen organization processes.

(H) Representative images of mIHC staining for APOD+ myCAFs in post-treatment samples. White asterisks highlight APOD+ myCAFs. Scale bar, 50 μm.

(I) Cell density of APOD+ myCAFs in baseline and post-treatment samples from mono-immunotherapy non-responders ($n = 9$), mono-immunotherapy responders ($n = 4$), and immunochemotherapy patients ($n = 6$). Horizontal bars indicate median. Two-sided unpaired t test; $*p < 0.05$; $**p < 0.01$; $***p < 0.001$.

(J) Representative images of mIHC staining for MYF5+ MSCs in post-treatment samples. White asterisks highlight MYF5+ MSCs. Scale bar, 50 μm.

(K) Cell density of MYF5+ MSCs in baseline and post-treatment samples from mono-immunotherapy non-responders ($n = 9$), mono-immunotherapy responders ($n = 4$), and immunochemotherapy patients ($n = 6$). Horizontal bars indicate median. Two-sided unpaired t test.

CellPress
OPEN ACCESS

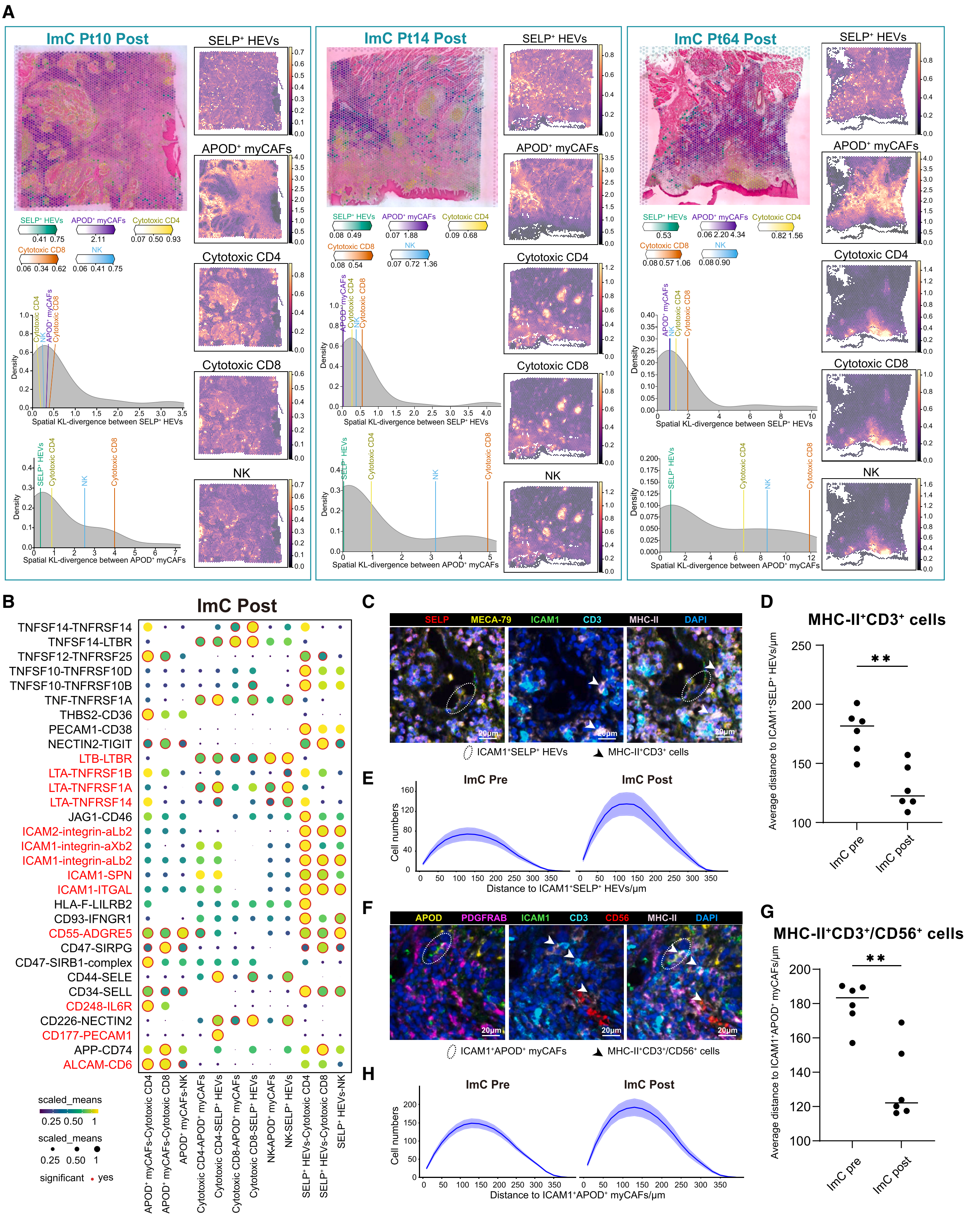

(legend on next page)

Additionally, cytotoxic T and NK cells displayed strong interactions with STMN1$^+$ cECs and MYF5$^+$ MSCs through PVR signaling pathways, with these immunosuppressive stromal niches particularly interacting with cytotoxic T cells via TIGIT signaling (Figure 7E). Spatial analysis further revealed elevated levels of ECM organization and degradation, particularly within tumor invasion regions, which were associated with a suppressed immune response and may adversely impact overall immune activity (Figure 7F) Collectively, these findings suggest that STMN1$^+$ cECs and MYF5$^+$ MSCs play critical roles in promoting immune resistance and facilitating tumor progression by mediating ECM remodeling and immunosuppressive interactions within the TME.

DISCUSSION

Immune checkpoint blockade therapies have ushered in a new era in the treatment of advanced cancers. However, only a few patients experience durable benefits from these therapies.[22,23] A significant challenge arises from the dynamic and heterogeneous nature of the TME, which evolves as cancer progresses. Stromal cells, including endothelial cells and fibroblasts, play a dominant role in shaping the TME structure and characteristics. These cells supply oxygen and nutrients and secrete ECM proteins. Emerging evidence also suggests that these two cell types have specific immune-modulating functions, indicating their complex roles in tumor immune evasion and response to immunotherapy.[24,25]

TECs often exhibit reduced expression levels of adhesion molecules such as ICAM1 and VCAM-1, which facilitate lymphocyte homing. Additionally, TECs can upregulate the expression of immune inhibitory molecules such as PD-L1.[26,27] This creates a barrier that prevents lymphocytes from efficiently infiltrating the tumor, thus weakening the overall immune response. In our present study, we found that patients with OSCC who did not respond to immunotherapy showed an increase in cECs within the TME. These cells were less able to bind to lymphocytes and were more likely to suppress the immune response via the TIGIT pathway. These findings suggest that combining AAT, which can help normalize the tumor vasculature, with immunotherapy could significantly improve patient outcomes by enhancing lymphocyte infiltration and their ability to attack the tumor. In fact, in a previously reported clinical trial, patients with locally advanced resectable OSCC who received camrelizumab and apatinib, an anti-angiogenic treatment targeting VEGFR, as neoadjuvant therapy demonstrated a high major pathological response (MPR) rate with an acceptable safety profile.[28]

The endothelial cell compartment within the TME is diverse, and certain subsets may benefit from immunotherapy. Recent studies have reported the presence of a specialized subset of endothelial cells that form vessel structures resembling HEVs within or near TLSs at tumor sites. These HEV-like vessels are equipped to facilitate the entry of lymphocytes into the tumor, play a crucial role in the formation of TLSs, and boost the body's immune surveillance against tumors.[29] The presence of strong antigens in the TME leads to the spontaneous development of HEVs in murine tumor models.[30,31] In our study, a significant number of patients treated with immunochemotherapy experienced complete remission, which was closely linked to increased tumor antigen release and subsequent expansion of specialized SELP$^+$ HEV-like endothelial cells. In addition, we found that enhancing ICAM1 signaling in these endothelial cells is key to attracting more immune cells to the tumor sites. Interestingly, the interaction between SELP$^+$ HEV-like endothelial cells and T/NK cells through the lymphotoxin (LTα/LTβ) pathways seems to encourage HEV formation and stimulate the development of TLS. Studies have shown that tumor necrosis factor (TNF)-α and lymphotoxins, mainly produced by activated lymphocytes and NK cells, are essential for the formation of tumor-associated HEVs in mouse models.[32] It has also been suggested that macrophages may help initiate the formation of HEVs through TNF signaling pathways.[33] In our study, we observed that cytotoxic T/NK cells triggered TNF pathways, leading to the formation of HEVs, and possibly improved immune cell infiltration into the tumor.

Cancer cells transform the surrounding connective tissue into CAFs. These CAFs actively remodel the ECM, a scaffold-like structure in the TME.[10] This remodeling is linked to lower survival rates and the reduced effectiveness of PD-1 blockade therapy.[34] Specifically, a type of CAF known as myCAFs, which are often found close to cancer cells, stand out because they express genes related to muscle and produce high levels of TGF-β, a substance that suppresses the immune system's ability to fight cancer.[35,36] Contrary to previous studies that explored the role of myCAFs in mediating resistance to immunotherapy, our study identified a distinct population of MYF5$^+$ MSCs associated with immunotherapy resistance. MSCs, or muscle stem cells, encompass various subtypes, one of which is characterized by high MYF5 expression, indicating that these MSCs are primed

Figure 5. Spatial transcriptomic analysis revealed immunomodulatory niches in OSCC post-immunochemotherapy

(A) Estimated cell abundance is depicted by color intensity for all cell types (top left) as well as individually for each cell type (top right). The density plots illustrate the Kullback-Leibler (KL) divergence comparing the observed values between two clusters against the null distribution (bottom left).

(B) CellPhoneDB illustrating the intensity of ligand-receptor interactions across cytotoxic T/NK cells and immunochemotherapy-friendly stromal cells.

(C) Representative image of ICAM1$^+$SELP$^+$ HEVs and MHC-II$^+$CD3$^+$ cells. Elliptical regions highlight ICAM1$^+$SELP$^+$ HEVs. White asterisks highlight MHC-II$^+$CD3$^+$ cells. Scale bar, 20 μm.

(D) Comparison of average distance from MHC-II$^+$CD3$^+$ cells to ICAM1$^+$SELP$^+$ HEVs pre- and post-treatment in immunochemotherapy responders. Horizontal bars indicate median. Two-sided unpaired t test; $^{**}p < 0.01$.

(E) Quantification of MHC-II$^+$CD3$^+$ cell numbers at varying radial distances from ICAM1$^+$SELP$^+$ HEVs.

(F) Representative image of ICAM1$^+$APOD$^+$ myCAFs and MHC-II$^+$CD3$^+$/CD56$^+$ cells. Elliptical regions highlight ICAM1$^+$APOD$^+$ myCAFs. White asterisks highlight MHC-II$^+$CD3$^+$/CD56$^+$ cells. Scale bar, 20 μm.

(G) Comparison of average distance from MHC-II$^+$CD3$^+$/CD56$^+$ cells to ICAM1$^+$APOD$^+$ myCAFs pre- and post-treatment in immunochemotherapy responders. Horizontal bars indicate median. Two-sided unpaired t test.

(H) Quantification of MHC-II$^+$CD3$^+$/CD56$^+$ cell numbers at varying radial distances from ICAM1$^+$APOD$^+$ myCAFs.

CellPress
OPEN ACCESS

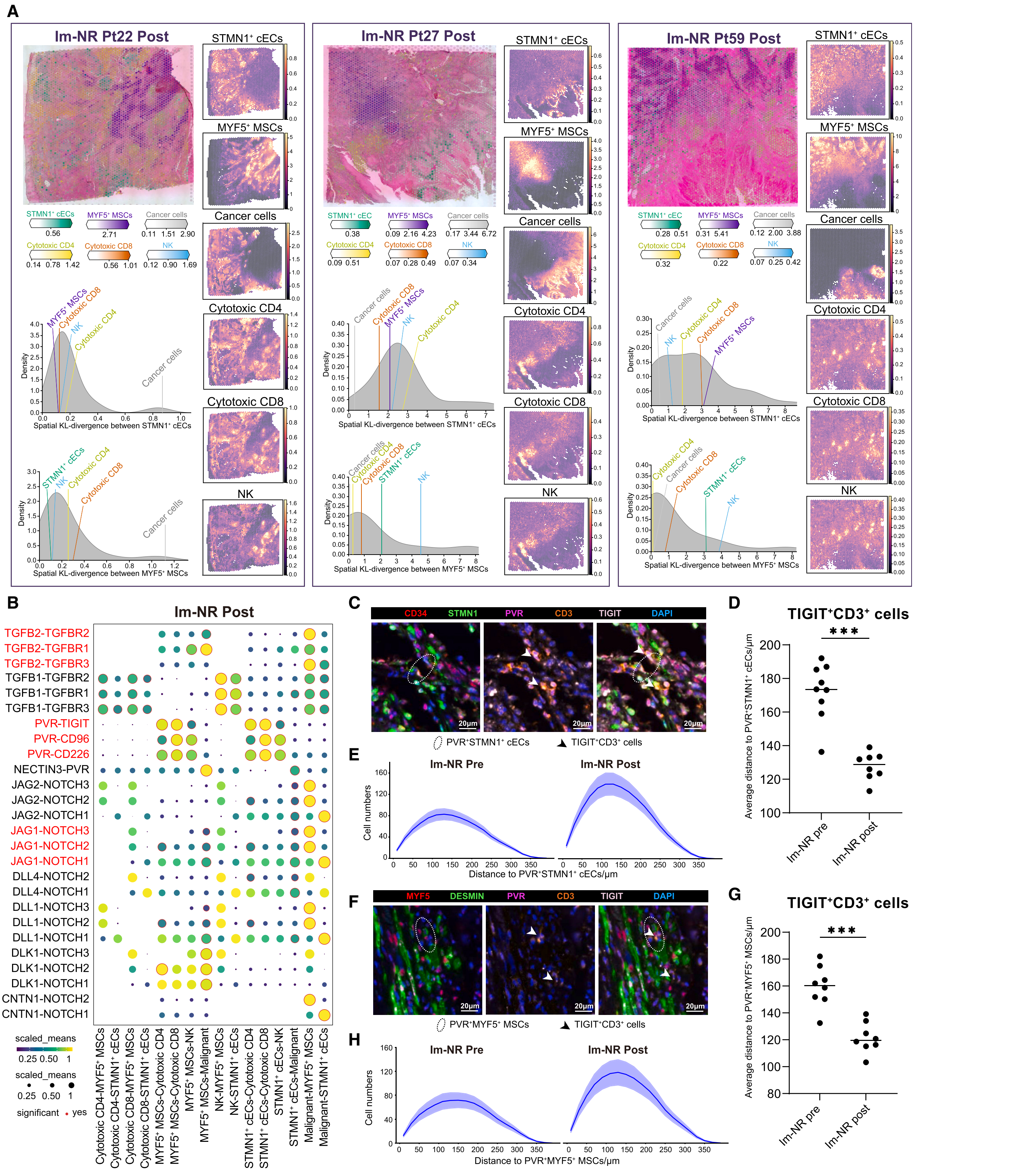

(legend on next page)

for early activation.[37] Previous research has suggested that MYF5$^+$ MSCs may contribute to the development of rhabdomyosarcoma.[38,39] Notably, the epithelial-to-mesenchymal transition (EMT) transcription factor Snail has been reported to regulate *MYF5* expression, thereby promoting rhabdomyosarcoma progression, indicating a potential link between tumor EMT processes and MYF5 expression.[40] However, the role of MYF5$^+$ MSCs within the TME of immunotherapy-resistant cancers remains unclear. In our analysis, the MYF5$^+$ MSCs were notably enriched at the invasive edges of tumors in patients resistant to mono-immunotherapy, particularly those with clinical disease progression. Furthermore, these cells interacted with tumor cells via TGF-β and NOTCH signaling pathways, contributing to ECM remodeling, while also suppressing immune responses through the PVR-TIGIT signaling axis. These dual roles in promoting tumor advancement and immune evasion suggest that MYF5$^+$ MSCs represent an underexplored stromal subset driving immunotherapy resistance, highlighting their potential as a therapeutic target warranting further investigation.

On the other hand, fibroblasts were also reported to play a crucial role in tumor-promoting inflammation.[41] It has been shown that immune-regulatory or inflammatory CAFs can foster cancer progression, facilitate immune evasion, and accelerate metastasis, largely mediated by the secretion of cytokines and chemokines. These factors recruit immunosuppressive myeloid cells, such as myeloid-derived suppressor cells and T regulatory cells, which further suppress the infiltration of cytotoxic lymphocytes and dendritic cells, and promote the polarization of macrophages toward an M2, pro-tumoral phenotype.[42–44] However, recent studies using scRNA-seq or novel markers have illuminated the heterogeneity of the CAF subpopulations. For instance, it has been reported that CAFs expressing complement regulatory factors enhance inflammation and immune response within the TME, which were observed proximal to pancreatic cancer cells but diminished as tumors progressed.[45] Moreover, CAFs stimulated by T cells can reshape the immunoregulatory landscape of OSCC. These CAFs mitigated CD8$^+$ T cell exhaustion, bolstered resident memory phenotypes, and amplified the cytolytic capabilities of T cells.[13] Recent research has also highlighted the prevalence of COL15A1$^+$ myCAFs in head and neck squamous cell carcinoma (HNSCC) patients, particularly in the peritumoral tissues across various cancers, where they are associated with tissue repair and regeneration.[46] These myCAFs show upregulation of genes related to adipogenesis, basement membrane components (such as *COL15A1*, *LAMA2*, and *COL18A1*), and tissue regeneration and proliferation (such as *APOD*, *APOE*, *IGF1*, and *MDK*), suggesting a role in maintaining epithelial cell regeneration and tissue homeostasis, consistent with their peri-epithelial location.[47] In our study, we observed an increase in APOD$^+$ myCAFs, which exhibit a gene expression profile similar to COL15A1$^+$ myCAFs among patients who responded positively to immunochemotherapy. These myCAFs exhibited strong interactions with cytotoxic T/NK cells through the ICAM1, CD55, CXCL13, and CXCL16 signaling pathways, suggesting a potential contribution to tumor regression.

Stromal cells, including endothelial cells and fibroblasts, interact with each other and other cell components, such as cancer cells and immune cells, creating a complex microenvironment ecosystem known as a niche. Using spatial transcriptomics, our study has revealed two distinct immunomodulatory niches within the tumor landscape that may be associated with the pathological response and durable survival outcomes of patients. The first niche, predominantly observed in patients responding positively to immunochemotherapy, is characterized by the notable presence of SELP$^+$ HEV-like vessels and a dense population of APOD$^+$ myCAFs within the tumor recovery zones. These anti-tumor stromal cells are not only intimately associated with TLS but also foster a TME that is conducive to effective immune responses, enhancing T cell penetration and antigen presentation. Conversely, the second niche, identified primarily in patients who did not respond to mono-immunotherapy, is distinguished by the proliferation of STMN1$^+$ cECs and a significant accumulation of MYF5$^+$ MSCs at the invasive frontiers of the tumor. This pro-tumor stromal configuration, aside from its close association with cancer cells, promotes an immunosuppressive TME by releasing inhibitory signals, particularly PVR, which engages with TIGIT on immune cells and suppresses their functions. Intriguingly, regions rich in STMN1$^+$ cECs and MYF5$^+$ MSCs also contained malignant cells with an activated NOTCH signaling pathway. This may indicate a potential for the relapse of these stem-like cancer cells, even among patients who initially responded to immunochemotherapy. However, long-term follow-up is critical for verifying this hypothesis. Going forward, research efforts should be directed toward remodeling these immunomodulatory stromal niches toward a more immune-friendly orientation, thus enhancing the efficacy of neoadjuvant immunotherapy strategies.

Figure 6. Spatial transcriptomic analysis revealed immunosuppressive niches in non-responders of OSCC post-immunotherapy

(A) Estimated cell abundance is depicted by color intensity for all cell types (top left) as well as individually for each cell type (top right). The density plots illustrate the Kullback-Leibler (KL) divergence comparing the observed values between two clusters against the null distribution (bottom left).

(B) Dot plot analysis showing significant coinhibitory interactions between cytotoxic T/NK cells and immunosuppressive stromal cells.

(C) Representative image of PVR$^+$STMN1$^+$ cECs and TIGIT$^+$CD3$^+$ cells. Elliptical regions highlight PVR$^+$STMN1$^+$ cECs. White asterisks highlight TIGIT$^+$CD3$^+$ cells. Scale bar, 20 μm.

(D) Comparison of average distance from TIGIT$^+$CD3$^+$ cells to PVR$^+$STMN1$^+$ cECs pre- and post-treatment in immunotherapy non-responders. Horizontal bars indicate median. Two-sided unpaired t test; ***$p < 0.001$.

(E) Quantification of TIGIT$^+$CD3$^+$ cell numbers at varying radial distances from PVR$^+$STMN1$^+$ cECs.

(F) Representative image of PVR$^+$MYF5$^+$ MSCs and TIGIT$^+$CD3$^+$ cells. Elliptical regions highlight PVR$^+$MYF5$^+$ MSCs. White asterisks highlight TIGIT$^+$CD3$^+$ cells. Scale bar, 20 μm.

(G) Comparison of average distance from TIGIT$^+$CD3$^+$ cells to PVR$^+$MYF5$^+$ MSCs pre- and post-treatment in immunotherapy non-responders. Horizontal bars indicate median. Two-sided unpaired t test.

(H) Quantification of TIGIT$^+$CD3$^+$ cell numbers at varying radial distances from PVR$^+$MYF5$^+$ MSCs.

CellPress
OPEN ACCESS

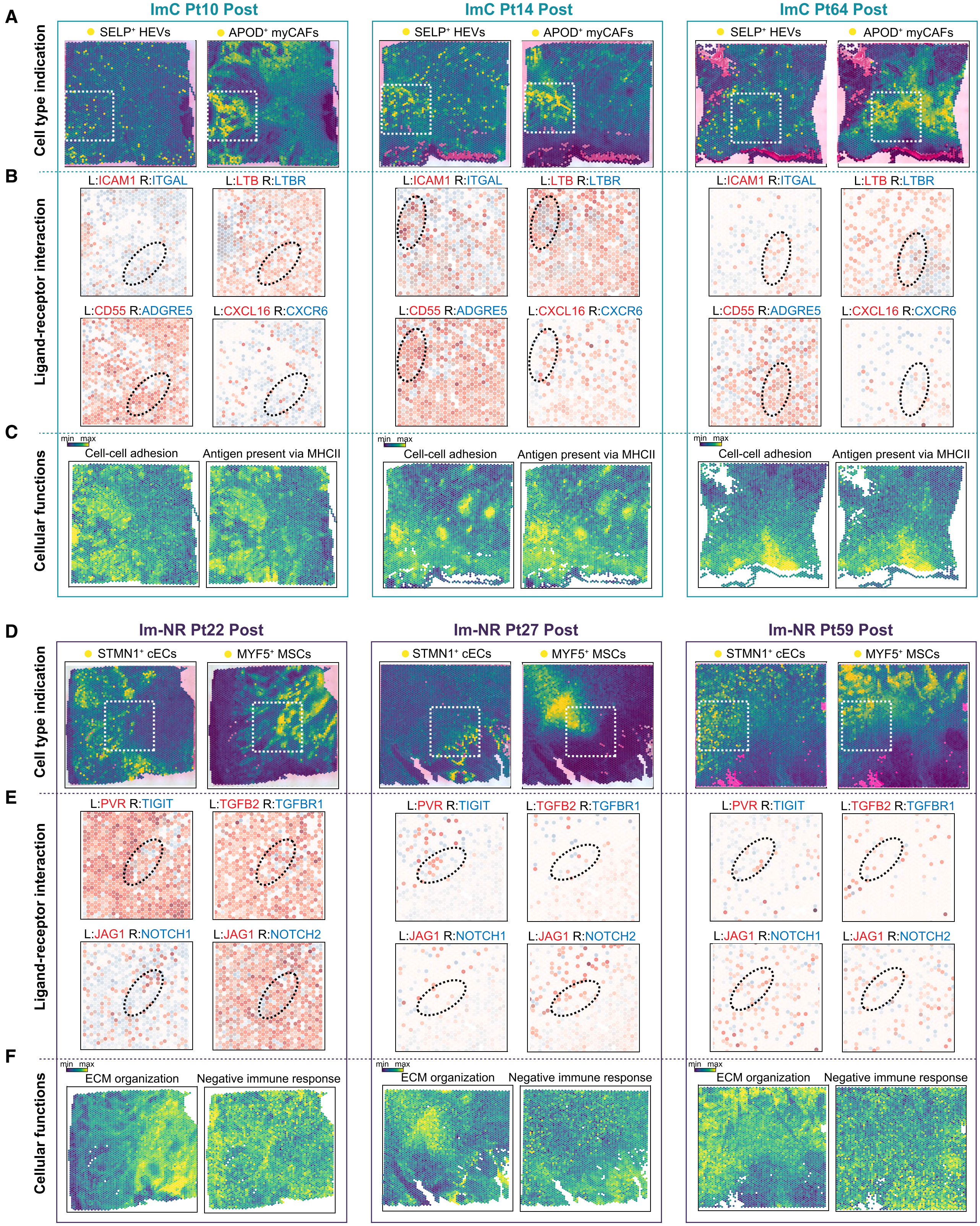

(legend on next page)

In summary, taking advantage of the two-arm design of our clinical trial, the present study revealed critical TSC subsets and their spatial-temporal dynamics during neoadjuvant immunotherapy and immunochemotherapy. We identified that the dynamic formation of immunostimulatory stromal niches, characterized by enrichment in SELP^{+} HEVs and APOD^{+} myCAFs, is associated with favorable responses to NAICT. In contrast, increased numbers of STMN1^{+} cECs and MYF5^{+} MSCs seem to promote immunosuppressive stromal niches, which are associated with poor outcomes in patients undergoing mono-immunotherapy.

Limitations of the study

One limitation of our study is that it is a single-center clinical trial with a relatively limited number of participants, which may restrict the generalizability of our findings. Additionally, our sample collection was primarily limited to pre-treatment biopsies and post-surgical specimens, lacking intermediate samples during the neoadjuvant treatment period, particularly after the first dose. This limitation prevents us to fully capture the dynamic changes in the TME. Furthermore, while our study offers valuable insights into clinical samples, it lacks in-depth functional validation, particularly regarding the potential therapeutic targets identified. Nonetheless, our study successfully identifies distinct tumor stromal niches in OSCC post-immunotherapy using single-cell and spatial transcriptomics.

RESOURCE AVAILABILITY

Lead contact

Further information and requests for resources and reagents should be directed to and will be fulfilled by the lead contact, Gang Chen (geraldchan@whu.edu.cn).

Materials availability

This study did not generate new unique reagents.

Data and code availability

- The raw sequence data reported in this paper have been deposited in the Genome Sequence Archive[48] in National Genomics Data Center[49], China National Center for Bioinformation/Beijing Institute of Genomics, Chinese Academy of Sciences, and are publicly accessible at https://ngdc.cncb.ac.cn/gsa-human. The single-cell data that support the findings of this study are available from GSA-Human (HRA005976). Spatial transcriptomic data are available from GSA-Human (HRA009391). All other relevant detailed clinical data could be available upon reasonable request from the lead contact.
- No custom computer codes are reported in this paper. The code for data analysis is available upon request.
- Any additional information required to reanalyze the data reported in this work paper is available from the lead contact upon request.

ACKNOWLEDGMENTS

This work was supported by the National Natural Science Foundation of China (82341023, 81922038, and 82272962); the Hubei Provincial Science and Technology Innovation Base (Platform) Project (2021CSA065); School and Hospital of Stomatology, Wuhan University (LYZX202001); the Interdisciplinary Research Project of School of Stomatology, Wuhan University (XNJC202305); the Innovative Research Team of High-level Local Universities in Shanghai (SHSMU-ZLCX20212300); and the Fundamental Research Funds for the Central Universities (2042022dx0003 and 2042023kfyq02).

AUTHOR CONTRIBUTIONS

Y.-T.L. and G.C. conceived the study. Y.-T.L., H.-M.L., J.-G.R., W.Z., and G.C. designed the study. Y.-T.L., H.-M.L., X.-X.W., Z.-L.Y., Q.-Y.F., X.-P.X., J.J., B.L., and W.Z. collected and analyzed the data. Y.-T.L., H.-M.L., and J.-G.R. drafted the manuscript. Y.-T.L., J.-G.R., W.Z., and G.C. wrote and revised the manuscript. G.C. supervised the study.

DECLARATION OF INTERESTS

The authors declare no competing interests.

STAR★METHODS

Detailed methods are provided in the online version of this paper and include the following:

- KEY RESOURCES TABLE
- EXPERIMENTAL MODELS AND STUDY PARTICIPANT DETAILS
- METHOD DETAILS
  - Tissue dissociation and preparation process
  - Reverse transcription, amplification, and library formation
  - Initial processing of scRNA-seq raw data
  - Analysis of differentially expressed genes and cell type identification
  - Gene set enrichment and gene set variation analysis
  - Cellular trajectory analysis
  - Analysis of intercellular communication
  - Generation of spatial transcriptomics data
  - Cell type deconvolution based on scRNA-seq data
  - Cell type co-localization analysis
  - Multiplex immunohistochemistry (mIHC) staining
- QUANTIFICATION AND STATISTICAL ANALYSIS
- ADDITIONAL RESOURCES

SUPPLEMENTAL INFORMATION

Supplemental information can be found online at https://doi.org/10.1016/j.xcrm.2025.102024.

Received: May 5, 2024
Revised: November 19, 2024
Accepted: February 19, 2025
Published: March 18, 2025

Figure 7. Spatial cell-cell crosstalk in tumor tissues from immunochemotherapy responders and mono-immunotherapy non-responders
(A) Spatial feature plots highlighted regions with high densities of SELP^{+} HEVs and APOD^{+} myCAFs subsets.
(B) The gene expression of ligand-receptor (L-R) pairs was analyzed in each spatial transcriptome slide. Elliptical regions were identified as areas with high levels of L-R communication within stromal interaction zones.
(C) Spatial feature plots showing the scores of functional signatures in various spatial transcriptomics (ST) slides.
(D) Spatial feature plots highlighted regions with high densities of STMN1^{+} cECs and MYF5^{+} MSCs subsets.
(E) The gene expression of ligand-receptor (L-R) pairs was analyzed in each spatial transcriptome slide. Elliptical regions were identified as areas with high levels of L-R communication within stromal interaction zones.
(F) Spatial feature plots showing the scores of functional signatures in various ST slides.

REFERENCES

1. Potente, M., and Mäkinen, T. (2017). Vascular heterogeneity and specialization in development and disease. Nat. Rev. Mol. Cell Biol. *18*, 477–494. https://doi.org/10.1038/nrm.2017.36.
2. Chhabra, Y., and Weeraratna, A.T. (2023). Fibroblasts in cancer: Unity in heterogeneity. Cell *186*, 1580–1609. https://doi.org/10.1016/j.cell.2023.03.016.
3. de Visser, K.E., and Joyce, J.A. (2023). The evolving tumor microenvironment: From cancer initiation to metastatic outgrowth. Cancer Cell *41*, 374–403. https://doi.org/10.1016/j.ccell.2023.02.016.
4. Winge, M.C.G., Kellman, L.N., Guo, K., Tang, J.Y., Swetter, S.M., Aasi, S.Z., Sarin, K.Y., Chang, A.L.S., and Khavari, P.A. (2023). Advances in cutaneous squamous cell carcinoma. Nat. Rev. Cancer *23*, 430–449. https://doi.org/10.1038/s41568-023-00583-5.
5. Hingorani, S.R. (2023). Epithelial and stromal co-evolution and complicity in pancreatic cancer. Nat. Rev. Cancer *23*, 57–77. https://doi.org/10.1038/s41568-022-00530-w.
6. Veneziani, A.C., Gonzalez-Ochoa, E., Alqaisi, H., Madariaga, A., Bhat, G., Rouzbahman, M., Sneha, S., and Oza, A.M. (2023). Heterogeneity and treatment landscape of ovarian carcinoma. Nat. Rev. Clin. Oncol. *20*, 820–842. https://doi.org/10.1038/s41571-023-00819-1.
7. Xu, M., Zhang, T., Xia, R., Wei, Y., and Wei, X. (2022). Targeting the tumor stroma for cancer therapy. Mol. Cancer *21*, 208. https://doi.org/10.1186/s12943-022-01670-1.
8. Jayson, G.C., Kerbel, R., Ellis, L.M., and Harris, A.L. (2016). Antiangiogenic therapy in oncology: current status and future directions. Lancet *388*, 518–529. https://doi.org/10.1016/S0140-6736(15)01088-0.
9. Garcia, J., Hurwitz, H.I., Sandler, A.B., Miles, D., Coleman, R.L., Deurloo, R., and Chinot, O.L. (2020). Bevacizumab (Avastin®) in cancer treatment: A review of 15 years of clinical experience and future outlook. Cancer Treat Rev. *86*, 102017. https://doi.org/10.1016/j.ctrv.2020.102017.
10. Caligiuri, G., and Tuveson, D.A. (2023). Activated fibroblasts in cancer: Perspectives and challenges. Cancer Cell *41*, 434–449. https://doi.org/10.1016/j.ccell.2023.02.015.
11. Wang, R., Song, S., Qin, J., Yoshimura, K., Peng, F., Chu, Y., Li, Y., Fan, Y., Jin, J., Dang, M., et al. (2023). Evolution of immune and stromal cell states and ecotypes during gastric adenocarcinoma progression. Cancer Cell *41*, 1407–1426.e9. https://doi.org/10.1016/j.ccell.2023.06.005.
12. Lambrechts, D., Wauters, E., Boeckx, B., Aibar, S., Nittner, D., Burton, O., Bassez, A., Decaluwé, H., Pircher, A., Van Den Eynde, K., et al. (2018). Phenotype molding of stromal cells in the lung tumor microenvironment. Nat. Med. *24*, 1277–1289. https://doi.org/10.1038/s41591-018-0096-5.
13. Obradovic, A., Graves, D., Korrer, M., Wang, Y., Roy, S., Naveed, A., Xu, Y., Luginbuhl, A., Curry, J., Gibson, M., et al. (2022). Immunostimulatory Cancer-Associated Fibroblast Subpopulations Can Predict Immunotherapy Response in Head and Neck Cancer. Clin. Cancer Res. *28*, 2094–2109. https://doi.org/10.1158/1078-0432.CCR-21-3570.
14. Li, J., Wu, C., Hu, H., Qin, G., Wu, X., Bai, F., Zhang, J., Cai, Y., Huang, Y., Wang, C., et al. (2023). Remodeling of the immune and stromal cell compartment by PD-1 blockade in mismatch repair-deficient colorectal cancer. Cancer Cell *41*, 1152–1169.e7. https://doi.org/10.1016/j.ccell.2023.04.011.
15. Cascone, T., Leung, C.H., Weissferdt, A., Pataer, A., Carter, B.W., Godoy, M.C.B., Feldman, H., William, W.N., Xi, Y., Basu, S., et al. (2023). Neoadjuvant chemotherapy plus nivolumab with or without ipilimumab in operable non-small cell lung cancer: the phase 2 platform NEOSTAR trial. Nat. Med. *29*, 593–604. https://doi.org/10.1038/s41591-022-02189-0.
16. Wu, H.-X., Pan, Y.-Q., He, Y., Wang, Z.-X., Guan, W.-L., Chen, Y.-X., Yao, Y.-C., Shao, N.-Y., Xu, R.-H., and Wang, F. (2023). Clinical Benefit of First-Line Programmed Death-1 Antibody Plus Chemotherapy in Low Programmed Cell Death Ligand 1–Expressing Esophageal Squamous Cell Carcinoma: A Post Hoc Analysis of JUPITER-06 and Meta-Analysis. J. Clin. Orthod. *41*, 1735–1746. https://doi.org/10.1200/JCO.22.01490.
17. Pignata, S., Scambia, G., Schettino, C., Arenare, L., Pisano, C., Lombardi, D., De Giorgi, U., Andreetta, C., Cinieri, S., De Angelis, C., et al. (2023). Carboplatin and paclitaxel plus avelumab compared with carboplatin and paclitaxel in advanced or recurrent endometrial cancer (MITO END-3): a multicentre, open-label, randomised, controlled, phase 2 trial. Lancet Oncol. *24*, 286–296. https://doi.org/10.1016/S1470-2045(23)00016-5.
18. Choueiri, T.K., McDermott, D.F., Merchan, J., Bauer, T.M., Figlin, R., Heath, E.I., Michaelson, M.D., Arrowsmith, E., D'Souza, A., Zhao, S., et al. (2023). Belzutifan plus cabozantinib for patients with advanced clear cell renal cell carcinoma previously treated with immunotherapy: an open-label, single-arm, phase 2 study. Lancet Oncol. *24*, 553–562. https://doi.org/10.1016/S1470-2045(23)00097-9.
19. Zhang, Z., Wu, B., Peng, G., Xiao, G., Huang, J., Ding, Q., Yang, C., Xiong, X., Ma, H., Shi, L., et al. (2022). Neoadjuvant Chemoimmunotherapy for the Treatment of Locally Advanced Head and Neck Squamous Cell Carcinoma: A Single-Arm Phase 2 Clinical Trial. Clin. Cancer Res. *28*, 3268–3276. https://doi.org/10.1158/1078-0432.CCR-22-0666.
20. Huang, X., Liu, Q., Zhong, G., Peng, Y., Liu, Y., Liang, L., Hong, H., Feng, W., Yang, S., Zhang, Y., et al. (2022). Neoadjuvant toripalimab combined with gemcitabine and cisplatin in resectable locally advanced head and neck squamous cell carcinoma (NeoTGP01): An open label, single-arm, phase Ib clinical trial. J. Exp. Clin. Cancer Res. *41*, 300. https://doi.org/10.1186/s13046-022-02510-2.
21. Huang, Y., Sun, J., Li, J., Zhu, D., Dong, M., Dou, S., Tang, Y., Shi, W., Sun, Q., Zhao, T., et al. (2023). Neoadjuvant immunochemotherapy for locally advanced resectable oral squamous cell carcinoma: a prospective single-arm trial (Illuminate Trial). Int. J. Surg. *109*, 2220–2227. https://doi.org/10.1097/JS9.0000000000000489.
22. Wolchok, J.D., Chiarion-Sileni, V., Gonzalez, R., Grob, J.-J., Rutkowski, P., Lao, C.D., Cowey, C.L., Schadendorf, D., Wagstaff, J., Dummer, R., et al. (2022). Long-Term Outcomes With Nivolumab Plus Ipilimumab or Nivolumab Alone Versus Ipilimumab in Patients With Advanced Melanoma. J. Clin. Orthod. *40*, 127–137. https://doi.org/10.1200/JCO.21.02229.
23. Ribas, A., and Wolchok, J.D. (2018). Cancer immunotherapy using checkpoint blockade. Science *359*, 1350–1355. https://doi.org/10.1126/science.aar4060.
24. Lavie, D., Ben-Shmuel, A., Erez, N., and Scherz-Shouval, R. (2022). Cancer-associated fibroblasts in the single-cell era. Nat. Cancer *3*, 793–807. https://doi.org/10.1038/s43018-022-00411-z.
25. Zeng, Q., Mousa, M., Nadukkandy, A.S., Franssens, L., Alnaqbi, H., Alshamsi, F.Y., Safar, H.A., and Carmeliet, P. (2023). Understanding tumour endothelial cell heterogeneity and function from single-cell omics. Nat. Rev. Cancer *23*, 544–564. https://doi.org/10.1038/s41568-023-00591-5.
26. Khan, K.A., and Kerbel, R.S. (2018). Improving immunotherapy outcomes with anti-angiogenic treatments and vice versa. Nat. Rev. Clin. Oncol. *15*, 310–324. https://doi.org/10.1038/nrclinonc.2018.9.
27. Fukumura, D., Kloepper, J., Amoozgar, Z., Duda, D.G., and Jain, R.K. (2018). Enhancing cancer immunotherapy using antiangiogenics: opportunities and challenges. Nat. Rev. Clin. Oncol. *15*, 325–340. https://doi.org/10.1038/nrclinonc.2018.29.
28. Ju, W.T., Xia, R.H., Zhu, D.W., Dou, S.J., Zhu, G.P., Dong, M.J., Wang, L.Z., Sun, Q., Zhao, T.C., Zhou, Z.H., et al. (2022). A pilot study of neoadjuvant combination of anti-PD-1 camrelizumab and VEGFR2 inhibitor apatinib for locally advanced resectable oral squamous cell carcinoma. Nat. Commun. *13*, 5378. https://doi.org/10.1038/s41467-022-33080-8.
29. Vella, G., Hua, Y., and Bergers, G. (2023). High endothelial venules in cancer: Regulation, function, and therapeutic implication. Cancer Cell *41*, 527–545. https://doi.org/10.1016/j.ccell.2023.02.002.
30. Rodriguez, A.B., Peske, J.D., Woods, A.N., Leick, K.M., Mauldin, I.S., Meneveau, M.O., Young, S.J., Lindsay, R.S., Melssen, M.M., Cyranowski, S., et al. (2021). Immune mechanisms orchestrate tertiary lymphoid structures

in tumors via cancer-associated fibroblasts. Cell Rep. *36*, 109422. https://doi.org/10.1016/j.celrep.2021.109422.

31. Peske, J.D., Thompson, E.D., Gemta, L., Baylis, R.A., Fu, Y.-X., and Engelhard, V.H. (2015). Effector lymphocyte-induced lymph node-like vasculature enables naive T-cell entry into tumours and enhanced anti-tumour immunity. Nat. Commun. *6*, 7114. https://doi.org/10.1038/ncomms8114.

32. Blanchard, L., and Girard, J.-P. (2021). High endothelial venules (HEVs) in immunity, inflammation and cancer. Angiogenesis *24*, 719–753. https://doi.org/10.1007/s10456-021-09792-8.

33. Johansson-Percival, A., He, B., Li, Z.-J., Kjellén, A., Russell, K., Li, J., Larma, I., and Ganss, R. (2017). De novo induction of intratumoral lymphoid structures and vessel normalization enhances immunotherapy in resistant tumors. Nat. Immunol. *18*, 1207–1217. https://doi.org/10.1038/ni.3836.

34. Qi, J., Sun, H., Zhang, Y., Wang, Z., Xun, Z., Li, Z., Ding, X., Bao, R., Hong, L., Jia, W., et al. (2022). Single-cell and spatial analysis reveal interaction of FAP+ fibroblasts and SPP1+ macrophages in colorectal cancer. Nat. Commun. *13*, 1742. https://doi.org/10.1038/s41467-022-29366-6.

35. Dominguez, C.X., Müller, S., Keerthivasan, S., Koeppen, H., Hung, J., Gierke, S., Breart, B., Foreman, O., Bainbridge, T.W., Castiglioni, A., et al. (2020). Single-Cell RNA Sequencing Reveals Stromal Evolution into LRRC15+ Myofibroblasts as a Determinant of Patient Response to Cancer Immunotherapy. Cancer Discov. *10*, 232–253. https://doi.org/10.1158/2159-8290.CD-19-0644.

36. Deng, C.-C., Hu, Y.-F., Zhu, D.-H., Cheng, Q., Gu, J.-J., Feng, Q.-L., Zhang, L.-X., Xu, Y.-P., Wang, D., Rong, Z., and Yang, B. (2021). Single-cell RNA-seq reveals fibroblast heterogeneity and increased mesenchymal fibroblasts in human fibrotic skin diseases. Nat. Commun. *12*, 3709. https://doi.org/10.1038/s41467-021-24110-y.

37. Lai, Y., Ramírez-Pardo, I., Isern, J., An, J., Perdiguero, E., Serrano, A.L., Li, J., García-Domínguez, E., Segalés, J., Guo, P., et al. (2024). Multimodal cell atlas of the ageing human skeletal muscle. Nature *629*, 154–164. https://doi.org/10.1038/s41586-024-07348-6.

38. Zibat, A., Missiaglia, E., Rosenberger, A., Pritchard-Jones, K., Shipley, J., Hahn, H., and Fulda, S. (2010). Activation of the hedgehog pathway confers a poor prognosis in embryonal and fusion gene-negative alveolar rhabdomyosarcoma. Oncogene *29*, 6323–6330. https://doi.org/10.1038/onc.2010.368.

39. Wei, Y., Qin, Q., Yan, C., Hayes, M.N., Garcia, S.P., Xi, H., Do, D., Jin, A.H., Eng, T.C., McCarthy, K.M., et al. (2022). Single-cell analysis and functional characterization uncover the stem cell hierarchies and developmental origins of rhabdomyosarcoma. Nat. Cancer *3*, 961–975. https://doi.org/10.1038/s43018-022-00414-w.

40. Skrzypek, K., Kusienicka, A., Trzyna, E., Szewczyk, B., Ulman, A., Konieczny, P., Adamus, T., Badyra, B., Kortylewski, M., and Majka, M. (2018). SNAIL is a key regulator of alveolar rhabdomyosarcoma tumor growth and differentiation through repression of MYF5 and MYOD function. Cell Death Dis. *9*, 643. https://doi.org/10.1038/s41419-018-0693-8.

41. Erez, N., Truitt, M., Olson, P., Arron, S.T., and Hanahan, D. (2010). Cancer-Associated Fibroblasts Are Activated in Incipient Neoplasia to Orchestrate Tumor-Promoting Inflammation in an NF-κB-Dependent Manner. Cancer Cell *17*, 135–147. https://doi.org/10.1016/j.ccr.2009.12.041.

42. Kato, T., Noma, K., Ohara, T., Kashima, H., Katsura, Y., Sato, H., Komoto, S., Katsube, R., Ninomiya, T., Tazawa, H., et al. (2018). Cancer-Associated Fibroblasts Affect Intratumoral CD8+ and FoxP3+ T Cells Via IL6 in the Tumor Microenvironment. Clin. Cancer Res. *24*, 4820–4833. https://doi.org/10.1158/1078-0432.CCR-18-0205.

43. Chakravarthy, A., Khan, L., Bensler, N.P., Bose, P., and De Carvalho, D.D. (2018). TGF-β-associated extracellular matrix genes link cancer-associated fibroblasts to immune evasion and immunotherapy failure. Nat. Commun. *9*, 4692. https://doi.org/10.1038/s41467-018-06654-8.

44. Steele, N.G., Biffi, G., Kemp, S.B., Zhang, Y., Drouillard, D., Syu, L., Hao, Y., Oni, T.E., Brosnan, E., Elyada, E., et al. (2021). Inhibition of Hedgehog Signaling Alters Fibroblast Composition in Pancreatic Cancer. Clin. Cancer Res. *27*, 2023–2037. https://doi.org/10.1158/1078-0432.CCR-20-3715.

45. Chen, K., Wang, Q., Li, M., Guo, H., Liu, W., Wang, F., Tian, X., and Yang, Y. (2021). Single-cell RNA-seq reveals dynamic change in tumor microenvironment during pancreatic ductal adenocarcinoma malignant progression. EBioMedicine *66*, 103315. https://doi.org/10.1016/j.ebiom.2021.103315.

46. Gao, Y., Li, J., Cheng, W., Diao, T., Liu, H., Bo, Y., Liu, C., Zhou, W., Chen, M., Zhang, Y., et al. (2024). Cross-tissue human fibroblast atlas reveals myofibroblast subtypes with distinct roles in immune modulation. Cancer Cell *42*, 1764–1783.e10. https://doi.org/10.1016/j.ccell.2024.08.020.

47. Joseph, D.B., Henry, G.H., Malewska, A., Reese, J.C., Mauck, R.J., Gahan, J.C., Hutchinson, R.C., Malladi, V.S., Roehrborn, C.G., Vezina, C.M., and Strand, D.W. (2021). Single-cell analysis of mouse and human prostate reveals novel fibroblasts with specialized distribution and microenvironment interactions. J. Pathol. *255*, 141–154. https://doi.org/10.1002/path.5751.

48. Chen, T., Chen, X., Zhang, S., Zhu, J., Tang, B., Wang, A., Dong, L., Zhang, Z., Yu, C., Sun, Y., et al. (2021). The Genome Sequence Archive Family: Toward Explosive Data Growth and Diverse Data Types. Genom. Proteom. Bioinform. *19*, 578–583. https://doi.org/10.1016/j.gpb.2021.08.001.

49. CNCB-NGDC Members and Partners, Xue, Y., Bao, Y., Zhang, Z., Zhao, W., Xiao, J., He, S., Zhang, G., Li, Y., Zhao, G., et al. (2022). Database Resources of the National Genomics Data Center, China National Center for Bioinformation in 2022. Nucleic Acids Res. *50*, D27–D38. https://doi.org/10.1093/nar/gkab951.

50. Wolf, F.A., Angerer, P., and Theis, F.J. (2018). SCANPY: large-scale single-cell gene expression data analysis. Genome Biol. *19*, 15. https://doi.org/10.1186/s13059-017-1382-0.

51. Polański, K., Young, M.D., Miao, Z., Meyer, K.B., Teichmann, S.A., and Park, J.-E. (2020). BBKNN: fast batch alignment of single cell transcriptomes. Bioinformatics *36*, 964–965. https://doi.org/10.1093/bioinformatics/btz625.

52. Kleshchevnikov, V., Shmatko, A., Dann, E., Aivazidis, A., King, H.W., Li, T., Elmentaite, R., Lomakin, A., Kedlian, V., Gayoso, A., et al. (2022). Cell2location maps fine-grained cell types in spatial transcriptomics. Nat. Biotechnol. *40*, 661–671. https://doi.org/10.1038/s41587-021-01139-4.

53. Dura, B., Choi, J.-Y., Zhang, K., Damsky, W., Thakral, D., Bosenberg, M., Craft, J., and Fan, R. (2019). scFTD-seq: freeze-thaw lysis based, portable approach toward highly distributed single-cell 3′ mRNA profiling. Nucleic Acids Res. *47*, e16. https://doi.org/10.1093/nar/gky1173.

54. Martin, M. (2011). Cutadapt removes adapter sequences from high-throughput sequencing reads. EMBnet. j. *17*, 10–12. https://doi.org/10.14806/ej.17.1.200.

55. Dobin, A., Davis, C.A., Schlesinger, F., Drenkow, J., Zaleski, C., Jha, S., Batut, P., Chaisson, M., and Gingeras, T.R. (2013). STAR: ultrafast universal RNA-seq aligner. Bioinformatics *29*, 15–21. https://doi.org/10.1093/bioinformatics/bts635.

56. Liao, Y., Smyth, G.K., and Shi, W. (2014). featureCounts: an efficient general purpose program for assigning sequence reads to genomic features. Bioinformatics *30*, 923–930. https://doi.org/10.1093/bioinformatics/btt656.

57. Hie, B., Bryson, B., and Berger, B. (2019). Efficient integration of heterogeneous single-cell transcriptomes using Scanorama. Nat. Biotechnol. *37*, 685–691. https://doi.org/10.1038/s41587-019-0113-3.

CellPress
OPEN ACCESS

58. Badia-i-Mompel, P., Vélez Santiago, J., Braunger, J., Geiss, C., Dimitrov, D., Müller-Dott, S., Taus, P., Dugourd, A., Holland, C.H., Ramirez Flores, R.O., and Saez-Rodriguez, J. (2022). decoupleR: ensemble of computational methods to infer biological activities from omics data. Bioinform. Adv. *2*, vbac016. https://doi.org/10.1093/bioadv/vbac016.

59. Hänzelmann, S., Castelo, R., and Guinney, J. (2013). GSVA: gene set variation analysis for microarray and RNA-Seq data. BMC Bioinf. *14*, 7. https://doi.org/10.1186/1471-2105-14-7.

60. Qiu, X., Hill, A., Packer, J., Lin, D., Ma, Y.-A., and Trapnell, C. (2017). Single-cell mRNA quantification and differential analysis with Census. Nat. Methods *14*, 309–315. https://doi.org/10.1038/nmeth.4150.

61. Efremova, M., Vento-Tormo, M., Teichmann, S.A., and Vento-Tormo, R. (2020). CellPhoneDB: inferring cell–cell communication from combined expression of multi-subunit ligand–receptor complexes. Nat. Protoc. *15*, 1484–1506. https://doi.org/10.1038/s41596-020-0292-x.

STAR★METHODS

KEY RESOURCES TABLE

REAGENT or RESOURCE	SOURCE	IDENTIFIER
Antibodies		
DARC	Abcam	Cat# ab137044; RRID: AB_3669012
PNAd	BD	Cat# 553863; RRID: AB_395099
SELP	Abcam	Cat# ab6632; RRID: AB_2184964
ICAM1	Cell Signaling Technology	Cat# 67836; RRID: AB_2799738
CD3	Cell Signaling Technology	Cat# 85061; RRID: AB_2721019
MHC-II	Abcam	Cat# ab180779; RRID: AB_3669013
PDGFRα+β	Abcam	Cat# ab32570; RRID: AB_777165
APOD	Abcam	Cat# ab256496; RRID: AB_3669014
CD56	Cell Signaling Technology	Cat# 99746; RRID: AB_2868490
PVR	Abcam	Cat# ab267788; RRID: AB_3669016
PAN-CK	Cell Signaling Technology	Cat# 4545; RRID: AB_490860
CD34	Abclonal	Cat# A13929; RRID: AB_2760781
STMN1	Abcam	Cat# ab52630; RRID: AB_2197257
TIGIT	Abcam	Cat# ab243903; RRID: AB_2943164
MYF5	ThermoFisher	Cat# MA5-26654; RRID: AB_2724766
DESMIN	Abcam	Cat# ab227651; RRID: AB_3669017
Biological samples		
Pre-treatment biopsy samples	This study	N/A
Post-treatment resected samples	This study	N/A
Critical commercial assays		
Opal 6-plex manual detection kit	Akoya	Cat# NEL811001KT
Visium Spatial Gene Expression Slide Kit	10x Genomics	Cat# 1000188
Deposited data		
Single-cell sequencing data	This study	GSA-Human: HRA00597
Spatial transcriptomic data	This study	GSA-Human: HRA009391
Software and algorithms		
CeleScope	Singleron	https://github.com/singleron-RD/
Python v3.10.6	Python.org	www.python.org/
Scanpy v1.9.3	Wolf et al.[50]	https://github.com/theislab/scanpy
BBKNN v1.3.6	Polański et al.[51]	https://github.com/Teichlab/bbknn
cell2location 0.1	Kleshchevnikov et al.[52]	https://github.com/BayraktarLab/cell2location
GraphPad Prism v9.4.1	GraphPad Software, La Jolla California, USA	www.graphpad.com
Akoya Phenoptics inForm	Akoya	https://www.akoyabio.com
Other		
Akoya Vectra3 system	Akoya	N/A

EXPERIMENTAL MODELS AND STUDY PARTICIPANT DETAILS

This study was approved by the Ethics Committee of the Hospital of Stomatology, Wuhan University ([2020] Ethics No.2). Eligible participants, both male and female, were between the ages of 18 and 70 years and had no prior treatments. All had newly diagnosed, histologically confirmed oral squamous cell carcinoma, staged clinically as III-IVA (T1-2N1-2M0 or T3-4aN0-2M0, based on the 8th edition of the American Joint Committee on Cancer (AJCC) staging system). To rule out distant metastasis, all patients underwent chest CT and whole-body bone scintigraphy. Additionally, all participants had an ECOG performance status of 0–1, demonstrated

CellPress
OPEN ACCESS

normal major organ function, and were deemed capable of tolerating the prescribed treatment regimen. The primary endpoint was the pathological response rate. Secondary endpoints included radiological response, 2-year event-free survival (EFS) rate, 2-year overall survival (OS) rate and treatment-related adverse events (TRAEs). Written informed consent was obtained from all participants, ensuring voluntary involvement and the absence of financial incentives. We collected 40 tumor samples from patients with OSCC between March 2021 to September 2022. Diagnosis was confirmed by seasoned pathologists using a comprehensive approach that included clinical assessment, phenotype analysis, and immunohistochemistry. The radiological response was evaluated by radiographic examinations and defined by Response Evaluation Criteria in Solid Tumors (RECIST) version 1.1. In this clinical trial, patients were divided into two categories based on their pathological response: those showing a pathological response rate >50% were classified as responders, whereas those showing a pathological response rate less than 50% were classified as non-responders.

The main exclusion criteria were as follows: patients who had experienced immune-related adverse events; those who had previously received antibody or drug targeting T cells or immune checkpoint pathways; and those requiring systemic steroid treatment due to autoimmune diseases. The detailed inclusion and exclusion criteria could be found in supplemented Method S1. After screening, sixty-eight eligible patients were randomly assigned (1:1) to receive camrelizumab (arm Cam) or camrelizumab plus TPF chemotherapy (arm Cam+TPF).

METHOD DETAILS

Tissue dissociation and preparation process

After biopsy or surgical extraction, the tissues were immediately immersed in sCelLive Tissue Preservation Solution (Singleron) and maintained under icy conditions. The tissues were rinsed three times in Hank's Balanced Salt Solution (HBSS) prior to fine cutting. For enzymatic dissociation, the chopped tissues were treated with 3 mL of sCelLive Tissue Dissociation Solution (Singleron) and processed using the Singleron PythoN Tissue Dissociation System at 37°C for 15 min. The obtained cell mixture was filtered using a sterile 40 μm pore-sized strainer and then treated with an equal volume of GEXSCOPE RBC Lysis Buffer (Singleron). After centrifugation for 5 min at 300 × g at 4°C, the supernatant was discarded, and the cell sediment was carefully resuspended in PBS. Cell viability was evaluated under a microscope after Trypan Blue staining.

Reverse transcription, amplification, and library formation

In the single-cell RNA sequencing (scRNA-seq) process, we adjusted cell suspensions to 200,000 cells/mL in PBS and then processed them using the Singleron Matrix Single Cell Processing System. The system's microwell chip isolated barcode beads and captured mRNA. This step guided the progress to the synthesis of complementary DNA (cDNA) via reverse transcription and amplification using PCR. The synthesized cDNA was fragmented and prepared for next-generation sequencing by attaching sequencing adapters. Following the protocol of the GEXSCOPE Single Cell RNA Library Kit (Singleron Biotechnologies),[53] the libraries were constructed, diluted to a concentration of 4 nM, combined, and finally subjected to sequencing on the Illumina NovaSeq 6000 platform, using 150 bp paired-end sequencing.

Initial processing of scRNA-seq raw data

Primary analysis of raw single-cell RNA sequencing (scRNA-seq) data was performed using the CeleScope pipeline (version 1.5.2, https://github.com/singleron-RD/CeleScope). To ensure quality, Cutadapt[54] (version 1.17) was applied to trim poly A tails and remove adapter sequences. This step also involved the extraction of cell barcodes and Unique Molecular Identifiers (UMIs). For the alignment of reads, the STAR[55] software (version 2.6.1a) was used and mapped them to the GRCh38 human reference genome (sourced from Ensembl release 92). Subsequently, FeatureCounts[56] (version 2.0.1) for the quantification of genes and UMI counts were instrumental in generating expression matrices for later stages of analysis (Table S2). Additionally, cells further selected with UMI counts below 30,000, gene counts within a range of 200 to 5,000, and mitochondrial DNA content less than 20%. Following these filtration steps, the Scanpy[50] software suite was used for more advanced analysis. We chose Scanorama[57] (version 1.7.4) for integrating scRNAseq data with ST data primarily to correct for potential batch effects that could arise, even among homogeneous tissue samples. By using both Scanorama and BBKNN[51] (version 1.3.6), we aimed to ensure that any technical variability across samples was minimized, thereby enhancing the accuracy of our spatial mapping. This approach allowed us to better explore the potential spatial distribution of phenotypes identified in the scRNAseq data within the tissue context. To identify malignancy, we assessed copy number variations (CNVs) using scRNA-seq data. This involved the interpretation of large-scale chromosomal CNVs in individual cells based on averaged expression profiles across chromosomal segments.

Analysis of differentially expressed genes and cell type identification

The 'rank_genes_groups' function within the Scanpy framework was used to pinpoint differentially expressed genes (DEGs) by applying the Wilcoxon rank-sum test with its standard settings. A gene was classified as differentially expressed if it appeared in more than 10% of the cells in any given cluster and had an average logarithmic (fold change) value above 0.25. To annotate cell types within each cluster, we integrated canonical markers found in DEGs with existing literature. The expression patterns of these markers were visualized using heatmaps and violin plots crafted through the 'pl.rank_genes_groups_heatmap' and 'pl.stacked_violin'

functions of Scanpy, respectively. The average *Z* score for each cluster was used to create heatmaps. Additionally, various cell types have been identified, including malignant cells (*KRT5*, *KRT7*, *KRT14*), endothelial cells (*CLDN5*, *FLT1, PECAM1, CDH5*), fibroblast cells (*COL1A1*, *DCN*, *C1R*), T cells (*CD3D*, *CD4*, *CD8A*), B cells (*CD79A*, *MS4A1*, *MZB1*), macrophages (*CD68*, *CD163*, *C1QA*), dendritic cells (*CD1C*, *CD1E*, *HLA-DQA1*), and mast cells (*CPA3*, *TPSAB1*, *TPSB2*). Furthermore, endothelial cells were classified into five main subtypes based on their unique gene expression profiles: venous endothelial cells (vECs; *CD52*), capillary endothelial cells (cECs; *RGCC, SPARC, SGK1, COL15A1*), arterial endothelial cells (aECs; *SEMA3G, HEY1, GJA5*), high endothelial venules (HEVs; *ACKR1, SELP*), and lymphatic endothelial cells (LECs; *PROX1, FTL4, PDPN*). Fibroblasts were similarly divided into five subtypes: myofibroblastic CAFs (myCAFs; *LRRC15, FAP,POSTN, COL1A1*), inflammatory CAFs (iCAFs; *CXCL8, IL6, CCL2*), antigen-presenting CAFs (apCAFs; *CD74, HLA-DRA*), smooth muscle cells (SMCs; *MYH11, MYLK, TAGLN*), and muscle satellite cells (MSCs; *MYF5, PAX7, DES*).

Gene set enrichment and gene set variation analysis

In our study, gene regulatory networks in single-cell RNA sequencing data were analyzed using DecoupleR[58] (version 1.4.0), a Python framework. DecoupleR utilizes the PROGENy method to convert changes in gene expression into pathway activity scores. ClusterProfiler (version 4.7.0) was used to determine biological and molecular functions. GSVA[59] (version 1.46.0) was used to calculate pathway activity scores according to calculate pathway activity scores for each cell, drawing on gene set enrichment analysis data from MsigDB (available at http://www.gsea-msigdb.org/gsea/msigdb/). The LIMMA package (version 3.54.0) was used to identify differences in the signaling pathways among the various groups. Pathways with adjusted *p* values greater than 0.05 were excluded from our analysis. Bar plots represent both the upregulated and downregulated pathways.

Cellular trajectory analysis

For the trajectory analysis of our combined dataset compiled with Seurat, we employed Monocle3 v.1.3.1[60] within RStudio. The initial step of the data processing involved the preprocess_cds function. Dimension reduction and cell clustering were performed using the reduce_dimension and cluster_cell functions, respectively. These processes have enabled the identification of cell clusters with similar gene expression profiles. Plot cell function is pivotal in visualization, arranging cells in a pseudo-time sequence. To further explore the developmental paths of the cells, we used the learning-graph function.

Analysis of intercellular communication

The CellPhoneDB[61] Python package (version 2.1.7) was used to explore and quantify potential communication networks between cell clusters. This package integrates the principles of molecular biology and bioinformatics. A CellPhoneDB object was created based on the mean expression levels of ligands, receptors, and associated cofactors in various cell groups. The normalized expression matrix was analyzed in detail. The cellphonedb plot dot_plot and cellphonedb plot heatmap_plot functions were used to visualize the intricate cell-to-cell communication network.

Generation of spatial transcriptomics data

Spatial transcriptomics was conducted on 11 samples, including five from immunochemotherapy patients (P10, P14, P62, P64, P66), one mono-immunotherapy responder (P13), and five mono-immunotherapy non-responders (P22, P27, P59, P65, P67), utilizing the advanced Visium spatial technology from 10× Genomics. For regions of interest (ROIs), we selected tumor restorative regions in immunochemotherapy patients and tumor invasive regions in mono-immunotherapy non-responders. RNA was meticulously extracted from 10 μm sections of FFPE tissue, using the Qiagen RNeasy FFPE Kit. RNA quality was assessed using an Agilent RNA 6000 Pico Kit. Blocks with DV200 values >50% were selected for additional processing. In the spatial capture stage, a single tissue section was carefully aligned in the capture zone of each Visium Spatial Gene Expression Slide Kit (PN-1000188, 10x Genomics). The tissue was subjected to a series of treatments, including deparaffinization, staining, and decrosslinking, followed by hybridization, ligation, release, and extension steps. Visium spatial gene expression FFPE libraries were meticulously assembled using both the Visium Human Transcriptome Probe Kit (PN-1000363) and Visium FFPE Reagent Kit (PN-1000363), according to the manufacturer's instructions. These libraries were then sequenced on the Illumina NovaSeq 6000 system, aiming for a depth of at least 75,000 read pairs per spot and identification of approximately 2,000 genes per spot. Further analysis of the spatial transcriptomic data was conducted using Scanpy.

Cell type deconvolution based on scRNA-seq data

Spatial transcriptomics data were processed using Spaceranger (v1.2.2). To map fibroblast and immune cell subsets in ST slides, we integrated 10× Visium data with scRNA-seq data through the cell2location package.[52] This approach estimates the abundance of each cell population at specific spatial locations by deconstructing mRNA counts in Visium data based on reference transcriptional profiles. Reference cell type signatures were trained using a negative binomial regression model within cell2location on scRNA-seq datasets. The model, trained with default parameters, provided posterior distributions of cell-type abundance for each spatial spot, facilitating downstream analysis. The cell type proportion for each specific cell type was then calculated as the proportion of spots with higher-than-average cell abundance across all selected spots on the ST slides.

CellPress
OPEN ACCESS

Cell type co-localization analysis

To analyze the spatial co-localization of stromal and immune cell subsets in spatial transcriptomics (ST) slides, we used spatial coordinates of clusters identified via the cell2location algorithm as inputs for a custom Python function that computed spatial density distributions of each cell subset using 30-bin histograms. For each cluster pair, we calculated the Kullback-Leibler (KL) divergence to quantify dissimilarities in spatial distributions. If histograms for any cluster pair were empty, the calculation was skipped, and NaN values were recorded. For valid pairs, density distributions were normalized, and KL divergence values were stored in a matrix. We visualized spatial dissimilarities using a KL divergence heatmap and a density plot of KL values. To determine statistical significance, we created a null distribution by shuffling spatial locations 1000 times and recalculating KL divergence for randomly selected groups representing 80% of the spots each time. Empirical *p* values were calculated as the fraction of permutations where the KL divergence exceeded the observed value, providing statistical validation of spatial co-localization patterns.

To identify ligand-receptor pairs and functional gene sets with elevated expression in favorable stromal subsets (SELP$^+$ HEVs and APOD$^+$ myCAFs), we computed signature scores and co-expression levels of ligand-receptor pairs within and outside ROIs. ROIs were defined as spatial locations where the estimated abundance of a specific cell subset exceeded the population average. Signature scores were calculated using the scanpy.tl.score_genes function, and co-expression scores for ligand-receptor pairs were determined by averaging the expression levels of the two genes in each pair. Statistical significance was assessed using the Wilcoxon rank-sum test. Similarly, we applied the ROI concept to unfavorable stromal subsets (STMN1$^+$ cECs and MYF5$^+$ MSCs) to evaluate ligand-receptor co-expression in these regions.

Multiplex immunohistochemistry (mIHC) staining

We collected 36 FFPE specimens from patients who had undergone single-cell RNA sequencing and performed immunofluorescence staining on these tumor sections to examine significantly altered patterns. The paraffin-embedded, paraformaldehyde-fixed tissues were sectioned into 2 μm slices. After deparaffinization and rehydration, the sections were re-fixed in 10% paraformaldehyde for 20 min. Multiplex immunohistochemistry (mIHC) was then conducted using the Opal 6-Plex Manual IHC Kit (NEL811001KT, Akoya Biosciences). To prepare for antibody incubation, tumor sections were blocked with Opal Antibody Diluent/Block for 10 min at room temperature, and then primary antibodies were applied for 1 h at 37°C. Following TBST and ddH$_2$O washes, sections were incubated with the secondary antibody (Opal Polymer HRP Ms + Rb) for 10 min at 37°C, rinsed in TBST and ddH$_2$O, and subsequently stained with a fluorescent dye (1:100 dilution in 1× Plus Amplification Diluent) for 10 min. DAPI (4′,6-diamidino-2-phenylindole) staining was then applied to all slides for 10 min at room temperature prior to imaging on the Akoya Vectra3 system. Finally, the images were processed and spectrally unmixed in sequence using Akoya Phenoptics inForm 1.0.2 software. The primary antibodies used for mIHC included: anti-human DARC (ab137044, Abcam, 1:200), PNAd (553863, BD, 1:400), SELP (ab6632, Abcam, 1:200), ICAM1 (67836S, CST, 1:200), CD3 (85061S, CST, 1:400), MHC-II (ab180779, Abcam, 1:200), PDGFRα+β (ab32570, Abcam, 1:400), APOD (ab256496, Abcam, 1:400), CD56 (99746S, CST, 1:200), PVR (ab267788, Abcam, 1:400), PAN-CK (4545S, CST, 1:800), CD34 (A13929, Abclonal, 1:200), STMN1 (ab52630, Abcam, 1:400), TIGIT (ab243903, Abcam, 1:200), MYF5 (MA5-26654, ThermoFisher, 1:400), and DESMIN (ab227651, Abcam, 1:400).

QUANTIFICATION AND STATISTICAL ANALYSIS

GraphPad Prism 9 (version 9.4.1, GraphPad Software) was used for statistical analysis. Comparisons of cell frequencies were performed using the nonparametric Wilcoxon rank-sum test. Additionally, two-sided t-tests, including paired and unpaired tests, were applied. *, $p < 0.05$; **, $p < 0.01$; ***, $p < 0.001$. $p < 0.05$ was considered statistically significant. Detailed statistical strategies and *p* values are provided in the relevant figures and their legends.

ADDITIONAL RESOURCES

This clinical trial has been registered on https://clinicaltrials.gov (NCT04649476).

Cell Reports Medicine, Volume *6*

Supplemental information

Immune-featured stromal niches associate with response to neoadjuvant immunotherapy in oral squamous cell carcinoma

Yu-Tong Liu, Hai-Ming Liu, Jian-Gang Ren, Wei Zhang, Xin-Xin Wang, Zi-Li Yu, Qiu-Yun Fu, Xue-Peng Xiong, Jun Jia, Bing Liu, and Gang Chen

Table S1. Clinical characteristics and response outcomes of OSCC patients undergoing immunotherapy. Related to Figure 1.

Patient ID	Gender	Age	Grade	Stage	Treatment	Pathologic response (tumor regression rate)	
1	Male	52	IVA	T4aN0M0	Im	pNR	0.00%
2	Male	56	III	T3N0M0	ImC	MPR	99.00%
3	Male	33	III	T2N1M0	Im	pNR	0.00%
4	Male	53	IVA	T3N2bM0	Im	MPR	93.00%
5	Female	63	IVA	T4aN0M0	Im	MPR	91.70%
6	Female	50	III	T3N0M0	ImC	pPR	62.55%
7	Male	52	IVA	T4aN2bM0	ImC	MPR	91.30%
8	Male	61	IVA	T3N2bM0	Im	pPR	67.13%
9	Male	49	IVA	T4aN0M0	ImC	MPR	97.89%
10	Male	48	IVA	T4aN2cM0	ImC	MPR	99.00%
11	Male	62	III	T3N0M0	Im	pNR	22.00%
12	Male	44	III	T3N0M0	Im	pNR	0.00%
13	Male	37	III	T3N0M0	Im	pPR	84.17%
14	Male	48	IVA	T3N2cM0	ImC	pPR	72.00%
15	Male	64	IVA	T4aN0M0	Im	pNR	0.00%
16	Male	63	III	T3N0M0	Im	pNR	33.00%
17	Male	67	III	T2N1M0	Im	MPR	99.00%
18	Male	65	III	T3N0M0	ImC	pCR	100.00%
19	Female	55	IVA	T4aN0M0	Im	pNR	0.00%
20	Male	39	IVA	T4aN1M0	ImC	MPR	97.49%
21	Male	52	IVA	T2N2bM0	ImC	MPR	99.00%
22	Female	66	III	T3N0M0	Im	pNR	28.16%
23	Male	65	III	T3N0M0	ImC	pPR	70.31%
24	Male	67	IVA	T4aN0M0	ImC	MPR	97.67%
25	Male	35	III	T3N0M0	ImC	MPR	97.41%
26	Male	33	IVA	T3N2cM0	ImC	MPR	99.00%
27	Female	64	III	T3N0M0	Im	pNR	0.00%
28	Male	50	IVA	T4aN0M0	Im	pNR	0.00%
29	Male	45	IVA	T3N2cM0	ImC	MPR	99.00%
30	Male	63	IVA	T4aN0M0	ImC	pCR	100.00%
31	Male	50	III	T3N0M0	ImC	pCR	100.00%
32	Male	34	IVA	T4aN0M0	Im	pNR	0.00%
33	Male	52	III	T3N0M0	Im	pPR	57.73%
34	Female	34	IVA	T3N2bM0	ImC	Non-evaluable	Non-evaluable
35	Male	65	IVA	T4aN0M0	Im	pNR	7.55%
36	Male	47	III	T2N1M0	ImC	MPR	90.00%
37	Male	65	III	T2N1M0	Im	pNR	0.00%
38	Male	42	III	T3N1M0	ImC	pCR	100.00%

39	Male	62	IVA	T4aN1M0	ImC	pPR	72.39%
40	Male	46	III	T3N0M0	Im	pNR	0.00%
41	Male	37	IVA	T2N2bM0	ImC	pNR	0.00%
42	Male	44	III	T3N0M0	ImC	MPR	99.00%
43	Male	38	III	T3N1M0	ImC	MPR	99.00%
44	Male	33	III	T3N1M0	Im	pNR	14.52%
45	Male	32	III	T3N0M0	Im	pPR	85.00%
46	Male	47	III	T3N0M0	Im	MPR	99.00%
47	Female	59	IVA	T4aN0M0	Im	pNR	0.00%
48	Male	52	IVA	T2N2bM0	Im	pNR	0.00%
49	Male	61	IVA	T2N2bM0	Im	pNR	0.00%
50	Female	44	III	T3N0M0	Im	pNR	0.00%
51	Male	66	III	T3N0M0	Im	pNR	0.00%
52	Male	39	III	T2N1M0	ImC	MPR	99.00%
53	Female	53	IVA	T2N2cM0	ImC	pCR	100.00%
54	Male	66	IVA	T2N2aM0	ImC	MPR	99.00%
55	Male	49	III	T3N1M0	ImC	Non-evaluable	Non-evaluable
56	Male	50	III	T3N1M0	ImC	pNR	0.00%
57	Male	52	IVA	T4aN2bM0	Im	pNR	0.00%
58	Male	36	III	T3N0M0	Im	pNR	0.00%
59	Male	66	III	T3N1M0	Im	pNR	32.33%
60	Male	50	III	T3N0M0	ImC	pCR	100.00%
61	Male	68	III	T3N0M0	ImC	pCR	100.00%
62	Male	46	III	T3N0M0	ImC	pCR	100.00%
63	Male	58	III	T2N1M0	ImC	pCR	100.00%
64	Male	36	IVA	T2N2aM0	ImC	MPR	99.00%
65	Male	42	III	T3N0M0	Im	pNR	0.00%
66	Male	48	III	T2N1M0	ImC	pCR	100.00%
67	Male	39	III	T3N0M0	Im	pNR	43.10%
68	Male	43	III	T2N1M0	Im	MPR	97.14%

Abbreviations: ImC, Immunochemotherapy; Im, Immunotherapy; pCR, pathologic complete response, MPR, major pathologic response; pPR, pathologic partial response; pNR, pathologic non-response.

Table S2. Summary of quality control (QC) metrics for individual patient samples. Related to Figure 2.

Patient ID	UMIs (median)	Genes	Genes (median)	Cell
10pre	8743	22603	2508	5606
10post	10626	23078	2560	2589
13pre	4903	23676	1635	5331
13post	4388	22537	1301	4842
14pre	6377	23861	2021	5366
14post	4341	23874	1472	8832
15pre	10368	25773	2873	6986
15post	4781	23506	1723	6735
16pre	6689	24008	2034	5186
16post	8662	25076	2617	5371
17pre	5935	24102	1588	3373
17post	4014	23255	1365	4115
18pre	6620	24584	2376	6889
18post	4944	22811	1625	3949
19pre	8256	25235	2538	9527
19post	6391	22471	2054	5074
22pre	2479	22020	901	5299
22post	4067	23130	1428	6958
27pre	4512	23329	1746	8085
27post	6337	21774	2124	3074
35pre	7494	25554	2484	8376
35post	4613	24345	1821	8352
37pre	4784	25173	1751	7882
37post	4133	25787	1460	7756
40pre	6717	24285	1760	7709
40post	9555	24550	2464	5038
49post	10909	24243	2813	6175
59post	4653	22148	1582	5223
62post	4349	22229	1425	6379
63post	4098	23523	1461	5568
64pre	12671	23410	2921	7049
64post	3463	22648	1288	4742
65pre	6793	23173	2124	3404
65post	9060	24278	2531	5072
66pre	2761	23203	1142	6363
66post	7020	23335	1840	5825
67pre	3142	21410	1217	4157
67post	3938	23093	1302	6763
68pre	5207	22279	1772	4220
68post	3948	21478	1362	5217

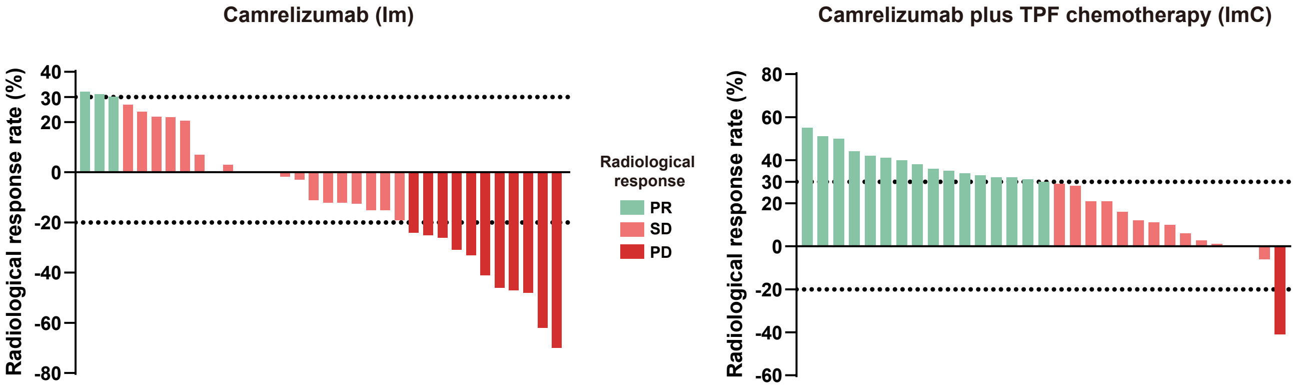

Figure S1. Waterfall plot showing patients' radiological response in arm Im and ImC. Related to Figure 1.

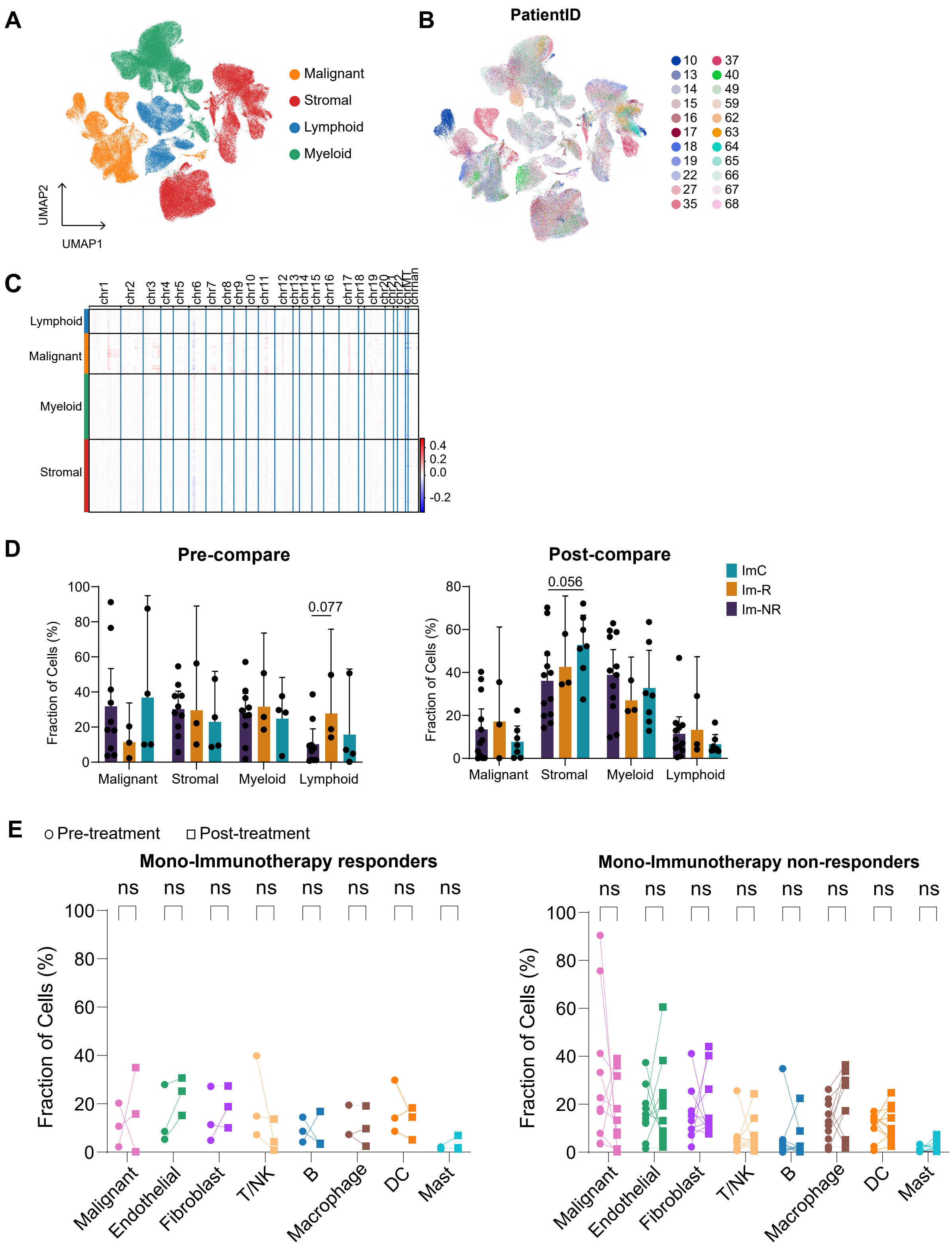

Figure S2. Comparison of cell type distribution before and after neoadjuvant immunotherapy in OSCC patients. Related to Figure 2. (A and B) UMAP plot categorizes all cells into malignant, stromal, lymphoid, myeloid types and showing the integration of all samples across patients in all clusters. (C) Heatmap depicting copy number variation (CNV) levels across different cell types in OSCC patients. (D) Stacked bar chart detailing the cell type composition for each patient pre- and post-immunotherapy treatment, grouped by response categories: ImC (Immunochemotherapy), Im-R (Immunotherapy response), and Im-NR (Immunotherapy non-response). Data is represented as mean ± SD. (E) Line plots illustrating the change in cellular fractions in paired samples pre- and post-immunotherapy treatment. The Wilcoxon rank-sum test tests statistical significance. *, $P < 0.05$; **, $P < 0.01$, ns, not significant.

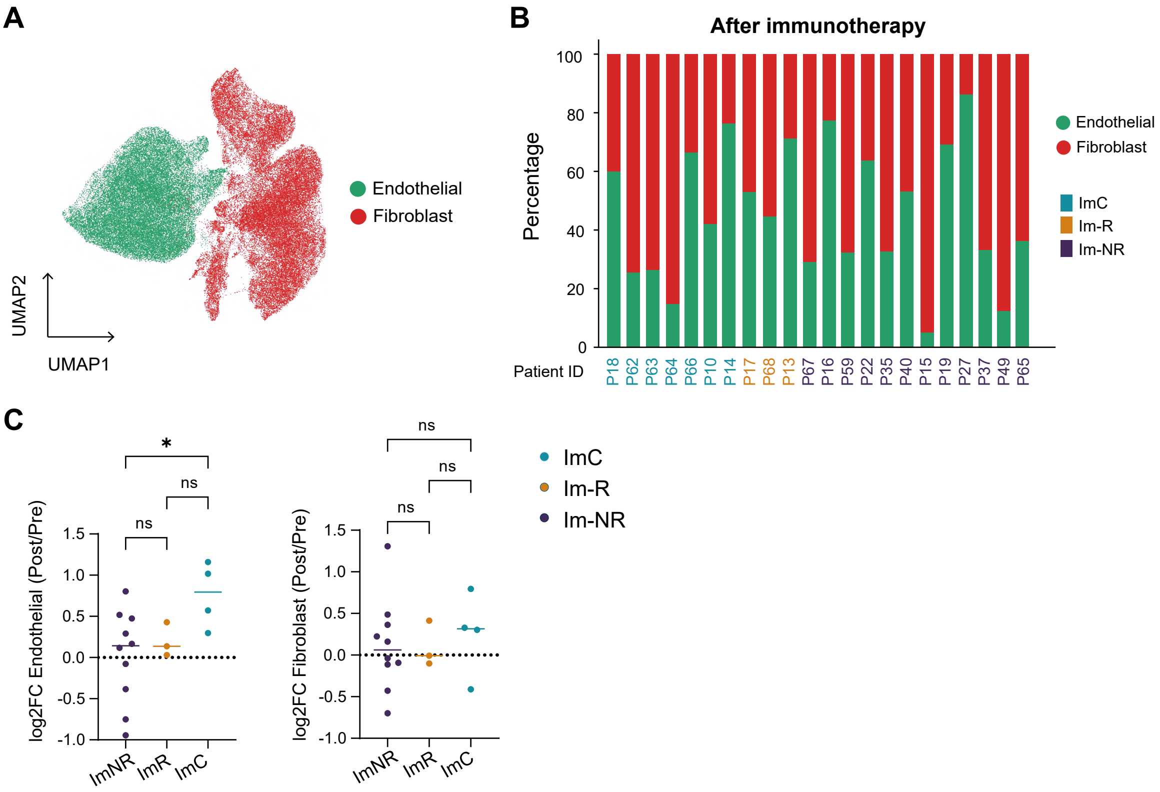

Figure S3. Comparison of post-immunotherapy stromal cell subsets in OSCC patients. Related to Figure 2.
(A) UMAP plot of stromal cells (n=81164) segregated by endothelial and fibroblast subsets. (B) Stacked bar chart illustrating the post-immunotherapy composition of endothelial and fibroblast cells across patients, with immunotherapy groups categorized as ImC (Immunochemotherapy), Im-R (Immunotherapy response), and Im-NR (Immunotherapy non-response). (C) Graphs showing log2 fold changes in endothelial and fibroblast subsets, grouped by immunotherapy response category. Horizonal bars indicate median. *, $P < 0.05$, ns, not significant.

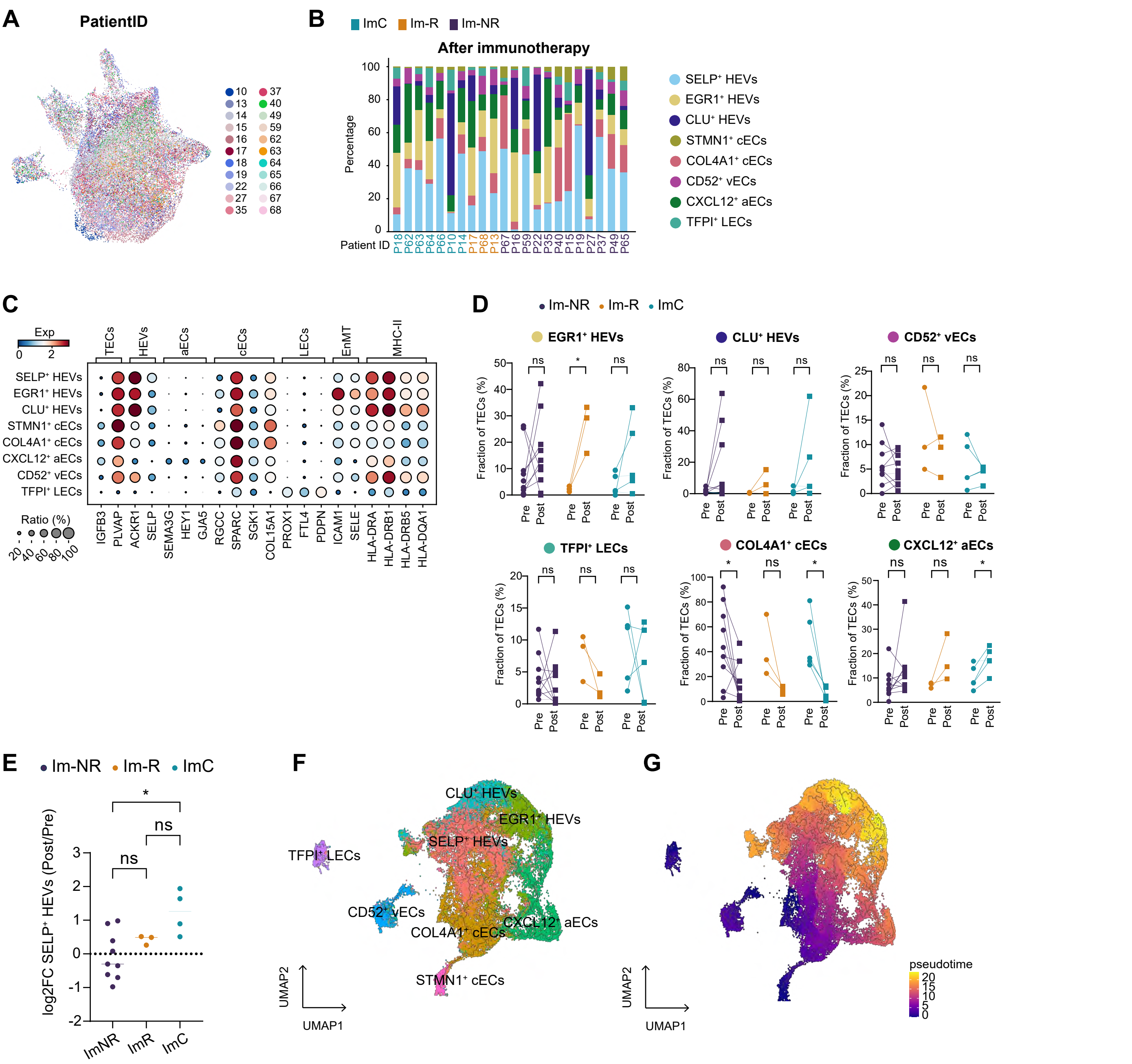

Figure S4. Endothelial cell subsets and dynamics before and after neoadjuvant immunotherapy in OSCC patients. Related to Figure 3. (A) UMAP plot showing the integration of all samples across patients in endothelial clusters. (B) The stacked bar chart depicting the post-immunotherapy distribution of endothelial cell subpopulations in OSCC patients, with immunotherapy groups categorized as ImC (Immunochemotherapy), Im-R (Immunotherapy response), and Im-NR (Immunotherapy non-response). (C) Dot plots showing the expression of signature genes across endothelial subtypes, with dot size and color intensity representing expression proportion and level, respectively. (D) Paired analysis graphs detailing the fraction of tumor endothelial cells (TECs) for specific subgroups ($EGR1^+$, CLU^+, $CD52^+$, $TFPI^+$, $COL4A1^+$, $CXCL12^+$) during immunotherapy. (E) Scatter plot showing log2 fold changes of $SELP^+$ HEVs in TME after immunotherapy grouped by treatment response. Horizonal bars indicate median. (F) UMAP plot color-coded by cell subtype revealing trajectory distribution. (G) UMAP projection displaying the progression of endothelial cell states over pseudotime, with colors transitioning from cold to warm to illustrate the developmental trajectory post-treatment. The Wilcoxon rank-sum test shows statistical significance. *, $P < 0.05$; **, $P < 0.01$; ns, not significant.

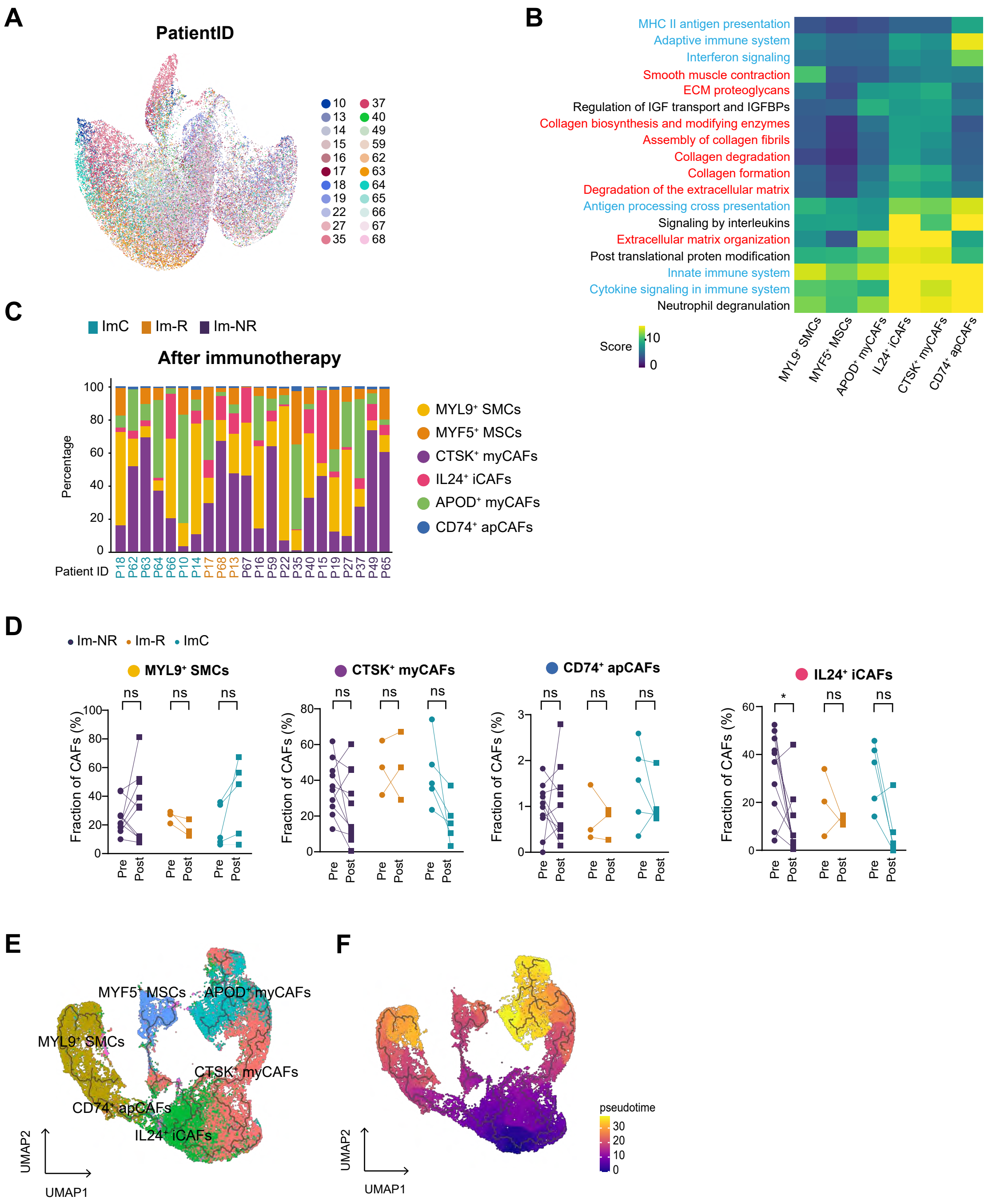

Figure S5. Fibroblast subsets and dynamics before and after neoadjuvant immunotherapy in OSCC patients. Related to Figure 4. (A) UMAP plot showing the integration of all samples across patients in fibroblast clusters. (B) Heatmap illustrating gene ontology enrichment scores for processes including antigen presentation and collagen organization. (C) A stacked bar chart illustrating the percentage of post-immunotherapy fibroblast subpopulations in OSCC patients, with immunotherapy groups categorized as ImC (Immunochemotherapy), Im-R (Immunotherapy response), and Im-NR (Immunotherapy non-response). (D) Line graphs displaying the change in the fraction of specific fibroblast subtypes (MYL9^{+}, CTSK^{+}, CD74^{+}, IL24^{+}) before (Pre) and after (Post) immunotherapy, with 'ns' indicating non-significant differences. (E) A UMAP plot delineating the distribution of fibroblast subtypes within the tumor microenvironment. (F) A UMAP projection depicting the developmental trajectory of fibroblast subtypes over pseudotime after immunotherapy, with the color gradient representing progression from initial to final states. The Wilcoxon rank-sum test shows statistical significance. *, $P < 0.05$; **, $P < 0.01$; ns, not significant.

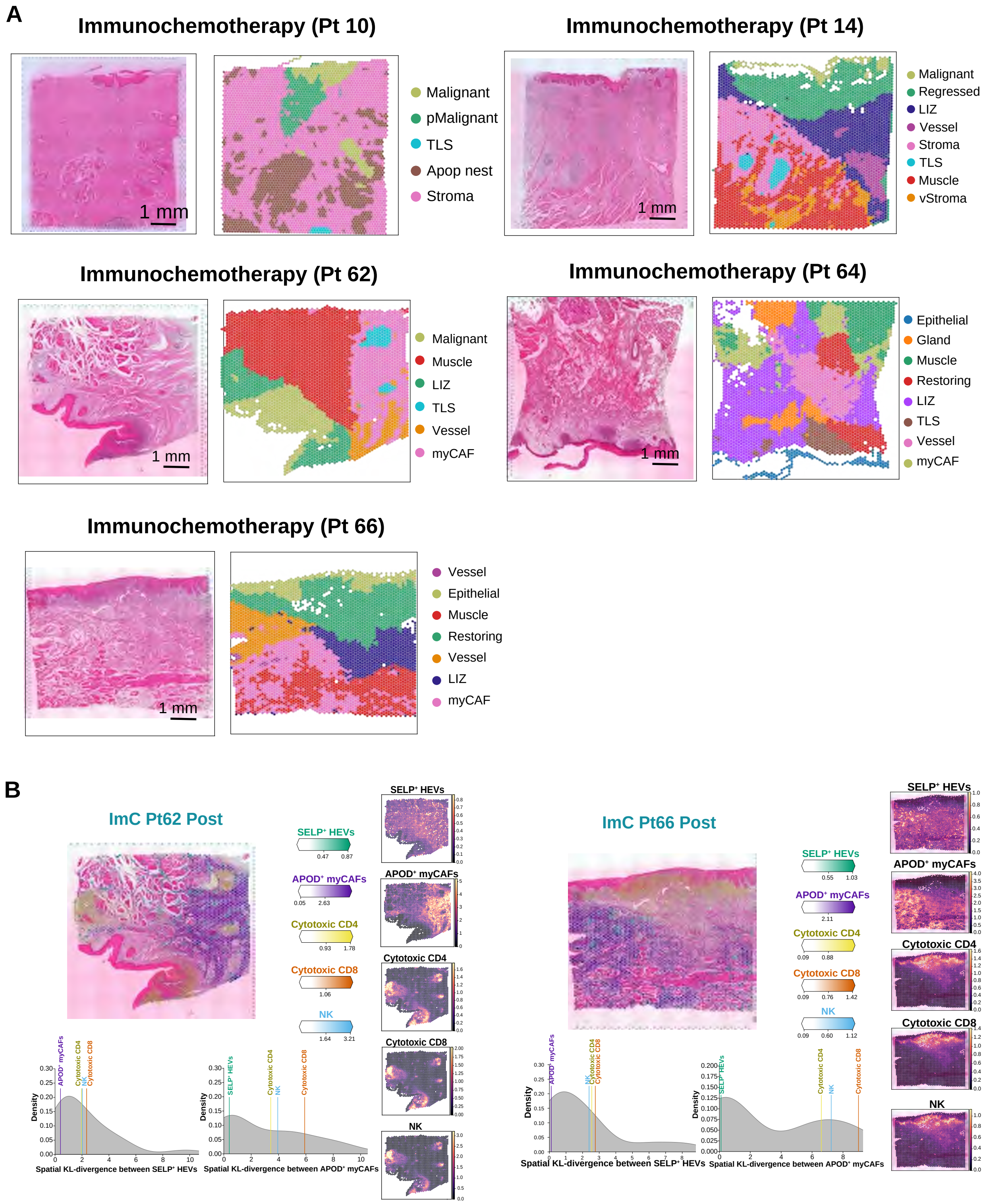

Figure S6. Overall landscapes of spatial transcriptomics in OSCC patients after immunochemotherapy. Related to Figure 5.
(A) Post immunochemotherapy (Patient #10, #14, #62, #64, #66) presenting a comparative view of histological tissue structure and a spatial transcriptomic map, identifying key cellular zones including epithelial region, malignant region, partial malignant region, regression region, restoring region, lymphoid cells infiltration zones (LIZ), vessels, stromal region, tertiary lymphoid structures (TLS), apoptosis nest, myCAF, gland, muscle fibers and vessels infiltration stroma (vStroma). Scale bar, 1 mm. (B) Estimated cell abundance is depicted by color intensity for interesting cell types (top left) as well as individually for each cell type (top right). The density plots illustrate the Kullback-Leibler (KL) divergence comparing the observed values between two clusters against the null distribution (bottom).

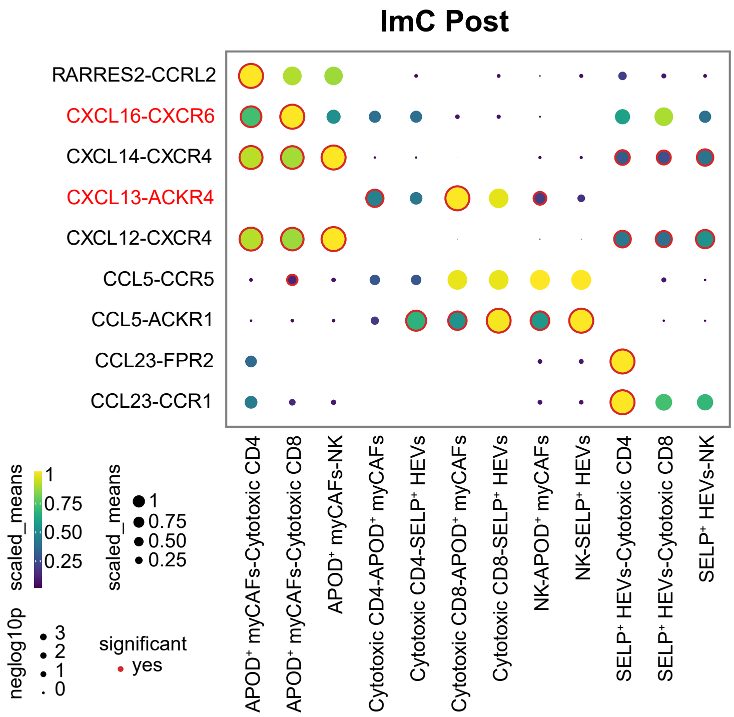

Figure S7. CellPhoneDB analysis of ligand-receptor of chemokine across cytotoxic cells and immunochemotherapy-friendly stromal cells in post-immunochemotherapy. Related to Figure 5.

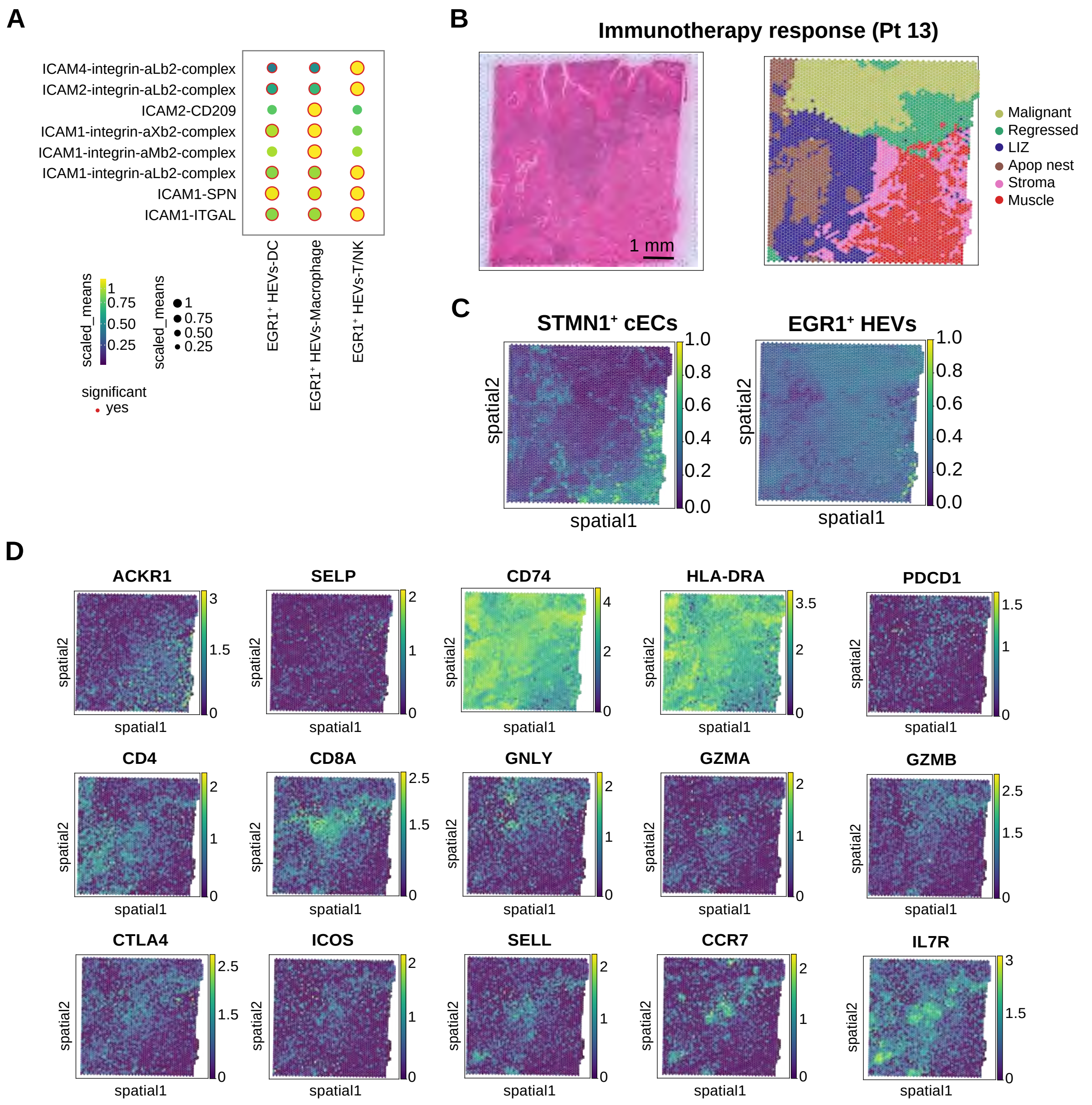

Figure S8. Spatial distribution of TEC subpopulations and T/NK markers in OSCC mono-immunotherapy responder. Related to Figure 5. (A) Dot plot matrix highlighting ICAM signaling interactions between EGR1^{+} HEVs and immune cells during immunotherapy. (B) Post immunochemotherapy (Patient #13) presenting a comparative view of histological tissue structure and a spatial transcriptomic map, identifying key cellular zones including malignant region, regression region, lymphoid cells infiltration zones (LIZ), apoptosis nest, stromal region, muscle. Scale bar, 1 mm. (C) Spatial heatmaps showing abundant STMN1^{+} cECs and EGR1^{+} HEVs in tumor regression regions. (D) The distribution of immune cell markers such as *ACKR1*, *CD4*, *CD8A*, cytotoxicity and antigen-presenting molecules were shown in tumor regression zones.

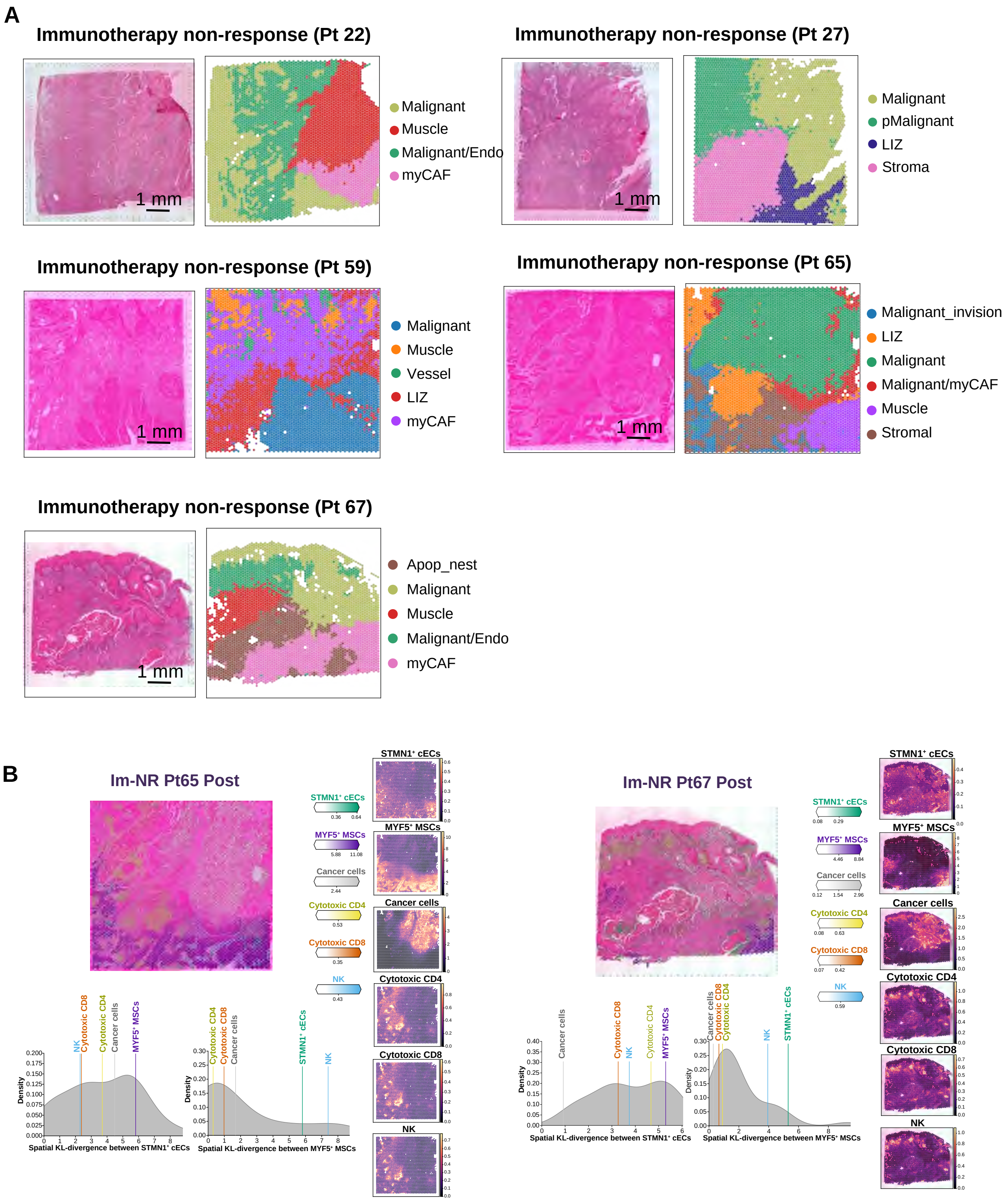

Figure S9. Overall landscapes of spatial transcriptomics in OSCC patients after immunotherapy. Related to Figure 6.
(A) Post immunotherapy non-responders (Patient #22, #27, #59, #65, #67) presenting a comparative view of histological tissue structure and a spatial transcriptomic map, identifying key cellular zones including malignant region, partial malignant region, apoptosis nest, lymphoid cells infiltration zones (LIZ), vessels, stromal region, muscle fibers and myCAF. Scale bar, 1 mm. (B) Estimated cell abundance is depicted by color intensity for interesting cell types (top left) as well as individually for each cell type (top right). The density plots illustrate the Kullback-Leibler (KL) divergence comparing the observed values between two clusters against the null distribution (bottom).

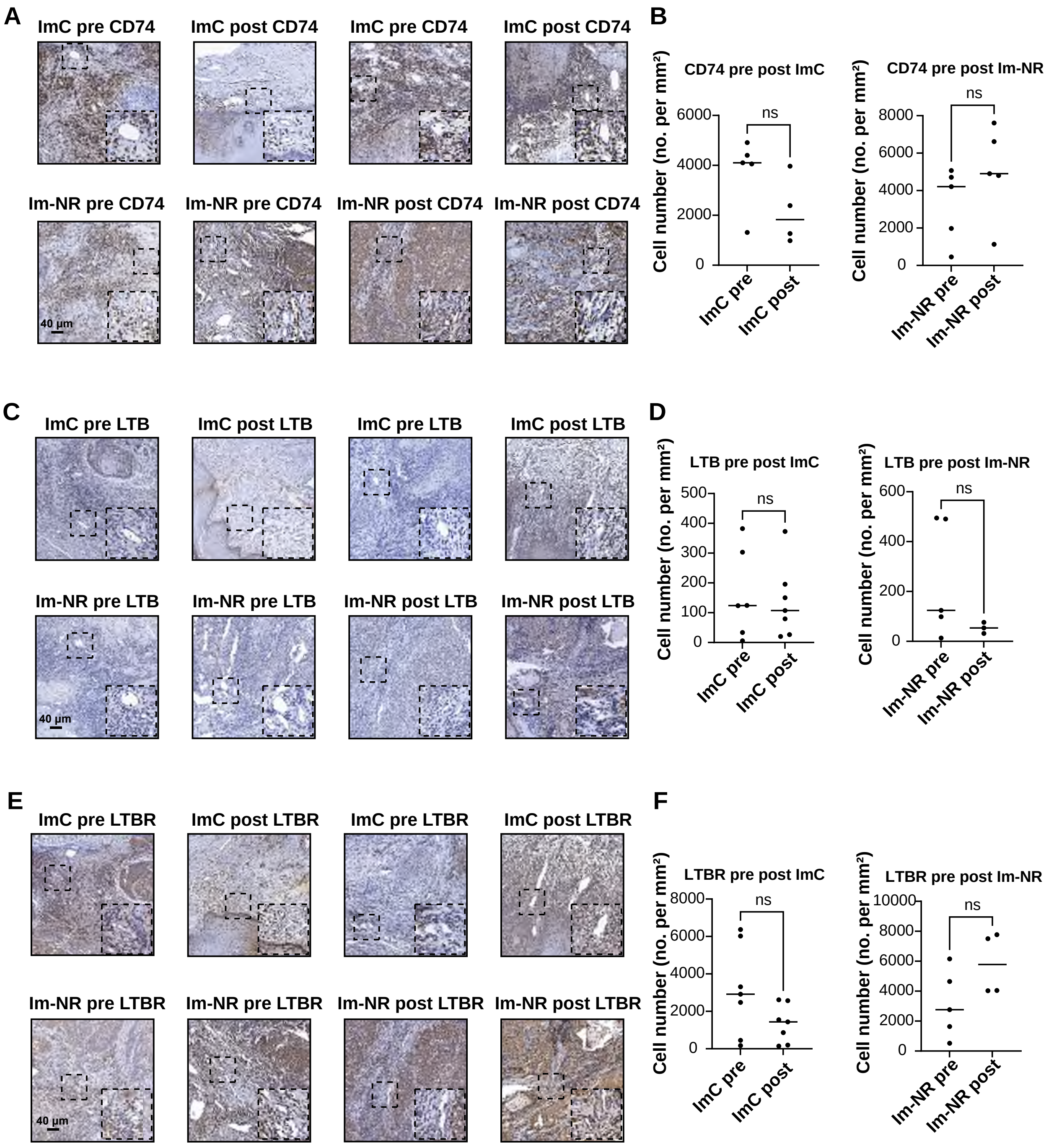

Figure S10. Immunohistochemical staining of LTB, LTBR and CD74 in OSCC patients undergoing immunotherapy and immunochemotherapy. Related to Figure 7.

(A) Immunohistochemical staining of pre-therapy and post-therapy CD74 in OSCC patients undergoing immunotherapy and immunochemotherapy. Scale bar, 40 μm. (B) Histogram of quantification of pre-therapy and post-therapy CD74 in OSCC patients undergoing immunotherapy and immunochemotherapy. Horizonal bars indicate median. (C) Immunohistochemical staining of pre-therapy and post-therapy LTB in OSCC patients undergoing immunotherapy and immunochemotherapy. Scale bar, 40 μm. (D) Histogram of quantification of pre-therapy and post-therapy LTB in OSCC patients undergoing immunotherapy and immunochemotherapy. Horizonal bars indicate median. (E) Immunohistochemical staining of pre-therapy and post-therapy LTBR in OSCC patients undergoing immunotherapy and immunochemotherapy. Scale bar, 40 μm. (F) Histogram of quantification of pre-therapy and post-therapy LTBR in OSCC patients undergoing immunotherapy and immunochemotherapy.
Horizonal bars indicate median.
